# Origin of thyrotropin-releasing hormone neurons that innervate the tuberomammillary nuclei

**DOI:** 10.1007/s00429-022-02527-5

**Published:** 2022-08-07

**Authors:** Edith Sánchez-Jaramillo, Gábor Wittmann, Judit Menyhért, Praful Singru, Gabriela B. Gómez-González, Eduardo Sánchez-Islas, Nashiely Yáñez-Recendis, Jaime Arturo Pimentel-Cabrera, Martha León-Olea, Balázs Gereben, Csaba Fekete, Jean-Louis Charli, Ronald M. Lechan

**Affiliations:** 1 Laboratorio de Neuroendocrinología Molecular, Dirección de Investigaciones en Neurociencias, Instituto Nacional de Psiquiatría Ramón de la Fuente Muñiz, 14370 México, CDMX México; 2 Tupper Research Institute and Department of Medicine, Division of Endocrinology, Diabetes, and Metabolism, Tufts Medical Center, Boston, MA 02111 USA; 3 Department of Endocrine Neurobiology, Institute of Experimental Medicine, Budapest, 1083 Hungary; 4 Departamento de Genética del Desarrollo y Fisiología Molecular, Instituto de Biotecnología, Universidad Nacional Autónoma de México (UNAM), 62210 Cuernavaca, MOR México; 5 Departamento de Neuromorfología Funcional, Dirección de Investigaciones en Neurociencias, Instituto Nacional de Psiquiatría Ramón de la Fuente Muñiz, 14370 México, CDMX México; 6 Laboratorio Nacional de Microscopia Avanzada, Instituto de Biotecnología, Universidad Nacional Autónoma de México (UNAM), 62210 Cuernavaca, MOR México; 7 Department of Neuroscience, Tufts University School of Medicine, Boston, MA 02111 USA; 8 Present Address: School of Biological Sciences, National Institute of Science Education and Research (NISER), Bhubaneswar, Odisha 752050 India

**Keywords:** TRH, Histamine, Retrograde, Anterograde, Lateral hypothalamus, *Trh–Cre* transgene

## Abstract

Hypophysiotropic thyrotropin-releasing hormone (TRH) neurons function as metabolic sensors that regulate the thyroid axis and energy homeostasis. Less is known about the role of other hypothalamic TRH neurons. As central administration of TRH decreases food intake and increases histamine in the tuberomammillary nuclei (TMN), and TMN histamine neurons are densely innervated by TRH fibers from an unknown origin, we mapped the location of TRH neurons that project to the TMN. The retrograde tracer, cholera toxin B subunit (CTB), was injected into the TMN E1–E2, E4–E5 subdivisions of adult Sprague–Dawley male rats. TMN projecting neurons were observed in the septum, preoptic area, bed nucleus of the stria terminalis (BNST), perifornical area, anterior paraventricular nucleus, peduncular and tuberal lateral hypothalamus (TuLH), suprachiasmatic nucleus and medial amygdala. However, CTB/pro-TRH178-199 double-labeled cells were only found in the TuLH. The specificity of the retrograde tract-tracing result was confirmed by administering the anterograde tracer, *Phaseolus vulgaris* leuco-agglutinin (PHAL) into the TuLH. Double-labeled PHAL-pro-TRH boutons were identified in all subdivisions of the TMN. TMN neurons double-labeled for histidine decarboxylase (*Hdc*)/PHAL, *Hdc*/*Trh receptor* (*Trhr*), and *Hdc*/*Trh*. Further confirmation of a TuLH-TRH neuronal projection to the TMN was established in a transgenic mouse that expresses Cre recombinase in TRH-producing cells following microinjection of a Cre recombinase-dependent AAV that expresses mCherry into the TuLH. We conclude that, in rodents, the TRH innervation of TMN originates in part from TRH neurons in the TuLH, and that this TRH population may contribute to regulate energy homeostasis through histamine *Trhr-*positive neurons of the TMN.

**Supplementary Information:**

The online version contains supplementary material available at 10.1007/s00429-022-02527-5.

## Introduction

Food intake, satiety and energy partition involve activity of hypothalamic neurons that integrate signals from the central and peripheral nervous systems and circulation. The humoral signals include nutrients such as glucose and hormones as leptin, insulin and ghrelin that control the activity of first-order sensory neurons in circumventricular organs, and of higher order neurons in the hypothalamus. Among the well-known neuroendocrine output of the brain are the hypophysiotropic neurons in the medial parvocellular region of the paraventricular nucleus (PVN) that produce thyrotropin-releasing hormone (TRH) and are involved in regulation of the thyroid axis, basal metabolism and thermogenesis (Ishikawa et al. 1988; Kawano et al. 1991; Fekete and Lechan 2014). In addition to its neurohormonal role in thyroid hormone homeostasis, however, TRH has other actions that impact energy balance. For example, the peripheral administration of TRH produces anorexia, an effect that is likely central since TRH administration into the cerebrospinal fluid or directly into the hypothalamus has a rapid, potent anorexigenic effect that is independent of thyroid function (Suzuki et al. 1982; Horita 1998).

Histamine neurons contribute to the anorexigenic output from the hypothalamus (Ookuma et al. 1989; Masaki and Yoshimatsu 2007; Panula and Nuutinen 2013). It has been proposed that histamine may mediate the anorexigenic effect of centrally administered TRH, as the effect can be attenuated by pretreatment with an irreversible inhibitor of L-histamine decarboxylase, the rate-limiting enzyme for histamine synthesis (Gotoh et al. 2007). In keeping with this observation is that histamine neurons, which are located exclusively in the hypothalamic tuberomammillary nuclei (TMN), are densely innervated by asymmetric TRH boutons (Sarvari et al. 2012), respond to TRH receptor agonists and are activated by TRH in vitro (Parmentier et al. 2009). To elucidate the origin of TRH neurons that innervate the TMN, we used a combination of retrograde and anterograde tracers and genetic and viral tools, identifying the tuberal lateral hypothalamic (TuLH) TRH neurons as an origin of the TRH input to the TMN.

## Materials and methods

### Animals

Adult male Sprague–Dawley rats (Taconic Farms, German Town, NY), 8 weeks old weighing 260–300 g from Tufts University and adult male and female C57BL/6NJ *Trh–Cre* mice from Instituto de Biotecnología-UNAM colony, 8 weeks old weighing 26–30 g were used in this study. Animals were acclimatized to standard environmental conditions (rats: lights on between 0600 and 1800 h, temperature 22 °C ± 1 °C, rat chow -2918 Envigo- and water ad libitum; mice: lights on between 0700 and 1900 h, temperature 22 °C ± 1 °C, water, and food -Harlan 2018SX- ad libitum). Protocols followed the NIH guide for the care and use of laboratory animals (8th ed.), and the Official Mexican Norm for production, care and use of laboratory animals NOM-062-ZOO-1999. All experimental protocols were reviewed and approved by the Institutional Animal Care and Use Committee at Tufts Medical Center and Tufts University School of Medicine and the institutional bioethics committee at Instituto de Biotecnología-UNAM and Instituto Nacional de Psiquiatría Ramón de la Fuente Muñiz. The number of animals, and number of sections per animal used in each experiment are shown in Table 1.

**Table 1:** Experimental design, protocols, mice and rat strains used

Protocol/section thickness	Gender/species	Region of interest (Bregma)	Number of animals with surgery	Number of animals with surgery in place	Number of animals used for ICC, ISH or ISH/ICC	Number of sections analyzed per animal for each series of sections (S1-S4)
Retrograde tract-tracing (CTB) (20 µm)	Male/Sprague–Dawley	vTMN (− 3.72 to − 4.92 mm)	19	10	6	S1, ICC CTB (*n* = 19 rats; *n* = 4 sections/rat); S2, ICC CTB/ rat pro-TRH_178-199_ (*n* = 10 rats; *n* = 4 sections/rat)
		dTMN (− 3.60 to − 3.84 mm)	6	4	4	S1, ICC CTB (n = 6 rats; *n* = 2 sections/rat); S2, ICC CTB/ rat pro-TRH_178-199_ (*n* = 4 rats; *n* = 2 sections/rat)
Anterograde tract-tracing (PHAL) (20 µm)	Male/Sprague Dawley	TuLH (− 1.72 to − 3.48 mm)	12	5	5	S1, ICC PHAL (*n* = 12 rats; *n* = 12 sections/rat); S2, ICC PHAL/ rat pro-TRH_178-199_ (*n* = 5 rats; *n* = 12 sections/rat)
Anterograde tract-tracing (PHAL) (20 µm)	Male/C57BL/ 6NJ	TuLH (− 1.06 to − 2.06 mm)	10	3	3	S1, ICC PHAL (*n* = 10 mice; *n* = 6 sections/mice); S2, ICC PHAL/ mouse pro-TRH_178-200_ (*n* = 3 mice; *n* = 6 sections/mice)
		TMN (− 2.18 to − 2.54 mm)				S3, ICC HDC/ PHAL (*n* = 3 mice; *n* = 6 sections/mice); S4, ISH *Hdc*/ ICC PHAL (*n* = 3 mice; *n* = 6 sections/mice)
Dual-label FISH (18 µm)	Male/Sprague Dawley	TuLH (− 1.72 to − 3.48 mm), TMN (− 3.60 to − 4.92 mm)	–	–	3	S1, S2 ISH *Trhr/Trh*, *Trhr2/Trh* (*n* = 8 sections/rat) S3, S4 ISH *Trhr/Hdc*, *Trhr2/Hdc* (*n* = 6 sections/rat)
(10 µm)	Male/C57BL/ 6NJ	TuLH (− 1.06 to − 2.06 mm)	–	–	3	S1 ISH *Trh*/*Cre* (*n* = 8 sections/mice)
(18 µm)	Male/C57BL/ 6NJ	TuLH (− 1.06 to − 2.06 mm)	–	–	3	S1 ISH *Trh*/*Cre* (*n* = 9 sections/mice)
		TMN (− 2.18 to − 2.54 mm)	–	–	3	S2 ISH *Trh*/*Hdc* (*n* = 8 sections/mice)
Transduction of *Trh* neurons with a Cre-recombinase–dependent AAV (20 µm)	Male AND female/*Trh–Cre* transgenic mice	TuLH, (− 1.06 to − 2.06 mm)	15	6	6	S1, ICC mCherry (*n* = 10 female, 5 male; *n* = 8 sections/mice); S2, ICC mCherry/ mouse pro-TRH_178-200_ (*n* = 4 female, 2 male; *n* = 8 sections/mice)
		TMN (− 2.18 to − 2.54 mm)				S3, ICC mCherry (*n* = 10 female, 5 male; *n* = 7 sections/mice)

### Retrograde tract-tracing experiments in rats

The retrograde tracer, cholera toxin ß subunit (CTB; List Biological Laboratories, Campbell, CA), was injected iontophoretically into the TMN, targeting E1–E2 subdivisions at stereotaxic coordinates from the bregma: AP = − 3.84 mm, DV = − 9.6 mm, *L* = − 1.5 mm or E4–E5 subdivisions at AP = − 3.84 mm, DV = − 9.33 mm, *L* = 0.33 mm (Paxinos and Watson 2009; Table 1). Animals were anesthetized i.p. with ketamine 50 mg/kg, xylazine 10 mg/kg body weight, and their heads mounted in a stereotaxic apparatus with bregma and lambda in the horizontal plane. A glass micropipette with 20 µm outer tip diameter filled with 0.5% CTB in distilled water was lowered into the brain through a burr hole. CTB was iontophoresed by a 6-µA positive current, pulsed on–off at 7-s intervals over 15 min, using a constant-current source (Stoelting, Wood Dale, IL). Post-operative analgesia was provided by administering buprenorphine, 0.05 mg/ kg subcutaneously. After a 10 day transport time, animals were deeply anesthetized with ketamine–xylazine and perfused transcardially with 20 ml phosphate-buffered saline (PBS; pH 7.4)-heparin, followed by 150 ml of 4% paraformaldehyde–0.1 M phosphate buffer (PB) at pH 7.4. The brains were removed, immersed in 30% sucrose for 1–2 days, and frozen on dry ice. Serial 25 µm-thick coronal sections were cut on a cryostat (Leica CM3050S, Nussloch GmbH, Germany) into one-in-six series of sections and collected into antifreeze solution (30% ethylene glycol; 25% glycerol; 0.05 M PB) to be stored at –20 °C until processed for immunohistochemistry. The positions of the injection sites were assessed by immunofluorescent staining. One series of sections were treated with 0.5% H_2_O_2_ and 0.5% Triton X-100 in PBS for 20 min, rinsed 3 times in 0.01 M PBS and incubated for 20 min in antibody diluent (2% normal horse serum, 0.2% Photoflo, 0.2% sodium azide in PBS) (Table 1). CTB injection sites were identified by overnight incubation of the sections in goat anti-CTB serum (#703, List Biological Laboratories) at 1:2,000 dilution, and subsequently in cyanine 3 (Cy3)-conjugated donkey anti-goat immunoglobulin G (IgG) for 2 h (Jackson Immunoresearch, 1:200 dilution). The specificity of the CTB antiserum was verified by the lack of any labeling in brain sections from animals that were not injected with CTB (Wittmann et al. 2009). Sections from brains with accurate injections into the TMN (Table 1) were further incubated overnight in a rabbit antibody against pro-TRH_178-199_ (a gift of Dr. E. Redei, Northwestern University, Chicago, IL), diluted at 1:50,000. This antiserum has been characterized by Nillni et al. (2001), who showed that it recognizes a 2.6 kDa peptide characteristic of prepro-TRH 178–199 in hypothalamic neurons in culture. Additionally, specificity for immunohistochemistry was assessed by preadsorbing the antiserum with the synthetic pro-TRH 178–199 peptide and showing no positive immunostaining in any of the preadsorption controls (Suzuki et al. 2001).

After washes in PBS, the sections were immersed in Alexa 488-conjugated donkey anti-rabbit IgG (Jackson Immunoresearch, 1:200 dilution) for 2 h. Sections were rinsed in PBS and once in Tris 0.05 M, pH 7.6, mounted onto glass slides and coverslipped with Vectashield antifade mounting medium with 4ʹ,6-diamidino-2-phenylindole, dihydrochloride (DAPI; Vector Laboratories) for imaging.

### Immunofluorescence imaging and data analysis (CTB experiments)

Immunofluorescence sections were observed using a Zeiss Axioplan 2 fluorescence microscope. The images were captured using a Spot digital camera (Diagnostic Instrument, Sterling Heights, MI), double exposed while switching filter sets for each fluorochrome, and superimposed in Adobe Photoshop CS4 using a Macintosh G4 computer to create a composite image of the same field. Filter sets included an Alexa 488 excitation filter of 450–490 nm, beam splitter of 495 nm, and emission filter of 500–550 nm; and Cy3 excitation filter of 538–562 nm, beam splitter of 570 nm, and emission filter of 570–640 nm.

Immunofluorescence sections with accurate injections into the TMN were further observed with a Zeiss 510 META laser confocal microscope (Carl Zeiss, Germany), using an Argon laser at 488 nm to visualize Alexa 488, and a Helium–Neon laser at 543 nm to visualize Cy3. The confocal microscope was attached to an Axiovert 200 M microscope with a 63X Plan Apo 1.4 oil objective (Carl Zeiss) for imaging. Prior to the analysis, a lambda stack was made to obtain the emission spectrum for each fluorophore, and barrier filters were placed to separate the fluorescence emission peak and avoid cross-linking of the emission spectrum curves [Alexa 488: excitation filter of 450–490 nm, beam splitter of 495 nm, and emission filter of 500–550 nm; Cy3, excitation of 538 –562 nm, beam splitter of 570 nm, and emission filter of 570–640 nm]. Confocal images were taken using the “multi-tracking” mode. The image acquisition parameters, such as the diameter of the pinhole, the gain detector, and the laser power, were adjusted to give the adjusted dynamic range in all observed preparations. A Z-stack was obtained from each section with optical cuts of 1 μm size in the Z axis. From the Z-stack, the image with the best immunoreactivity for TRH and CTB colocalization was chosen, and the Manders overlap coefficient analysis was determined with LSM 510, Carl Zeiss 4.0sp1 software. The value of one was considered as total colocalization as referenced by Manders et al. (1993).

### Anterograde tract-tracing experiments in rats

The anterograde tracer, *Phaseolus vulgaris* leuco-agglutinin (PHAL; Vector Laboratories, Burlingame, CA), was injected iontophoretically into the TuLH of 12 rats (Table 1) as follows. Rats were anesthetized i.p. with ketamine 50 mg/kg, xylazine 10 mg/kg body weight, and their heads positioned in a stereotaxic apparatus with bregma and lambda in the horizontal plane. A glass micropipette (20 µm outer tip diameter) filled with 2.5% PHAL in 0.01 M PB (pH 8.0) was lowered into the brain through a burr hole in the skull at the following stereotaxic coordinates from the bregma: AP =  − 2.92 mm, DV = − 9.4 mm, L =  − 1.6 mm corresponding to the TuLH (Paxinos and Watson 2009). The tracer was deposited by iontophoresis as described above. Rats were allowed to survive for 8 days and then deeply anesthetized with ketamine–xylazine and perfused transcardially with 20 ml 0.01 M PBS (pH 7.4), followed by 150 ml of 4% paraformaldehyde in 0.1 M PB, pH 7.4. The brains were rapidly removed, cut into two blocks, and cryoprotected by immersion in 30% sucrose-PBS overnight. The brains were sectioned at 25 µm into one-in-six series of sections and then collected into antifreeze solution to store at – 20 °C until used for immunohistochemistry. Sections were pre-treated with 0.5% H_2_O_2_ and 0.5% Triton X-100 in PBS for 20 min. Nonspecific antibody binding was reduced by treatment in 2% normal horse serum in PBS for 20 min after which they were incubated overnight in 1:5000 goat anti-PHAL antiserum (Vector labs) diluted in antibody diluent. After washing in PBS, the sections were incubated in 1:200 Cy3-conjugated donkey anti-goat IgG (Jackson Immunoresearch) for 2 h and rinsed in PBS. The specificity of PHAL antiserum was verified by the lack of any labeling in brain sections from animals that were not injected with PHAL (Supplemental Fig. 3, C1–C3). Sections from animals with injection sites in the TuLH area (Table 1) were further incubated in rabbit anti-pro-TRH_178-199_ antiserum (1:50,000, overnight), and subsequently in 1:400 Alexa 488-conjugated donkey anti-rabbit IgG (Jackson) for 2 h, at RT. Sections were rinsed and mounted as mentioned above. Antibody characterization has been described in detail (Wittmann et al. 2009).

### Anterograde tract-tracing experiments in mice

The anterograde tracer, PHAL, was injected iontophoretically into the TuLH of ten C57BL/6 J adult male mice as described for rats, except that the stereotaxic coordinates were from the bregma AP = − 1.94 mm, DV = − 5.5 mm, *L* = − 0.8 mm (Paxinos and Franklin 2001; Table 1). The tracer was deposited by iontophoresis, and mice were allowed to survive for 8 days and then deeply anesthetized with ketamine–xylazine, and perfused transcardially with 5 ml 0.01 M PBS (pH 7.4), followed by 50 ml of 3% paraformaldehyde in 0.1 M PB, pH 7.4/1% acrolein. The brains were rapidly removed, cryoprotected by immersion in 30% sucrose-PBS overnight, sectioned at 20 µm, and then either processed for PHAL/ histidine decarboxylase (HDC) floating immunohistochemistry or thaw-mounted on Fisherbrand Superfrost Plus Microscope Slides, air-dried, and stored at − 80 °C until used for double-labeling immuno-in situ hybridization (ISH) fluorescence.

For immuno-ISH fluorescence, a set of mounted sections from animals with injection sites in the TuLH area (*n* = 3; Table 1) were hybridized with a histidine decarboxylase (*Hdc*; a gift from Dr. Erik Hrabovszky) fluorescein 12-UTP riboprobe, thoroughly washed, dehydrated, and pre-treated with 0.5% H_2_O_2_ and 0.5% Triton X-100 in PBS to follow the immunodetection protocol. Double immunofluorescence was detected after incubating overnight in 1:5,000 goat anti-PHAL antiserum and 1:100 anti-mouse fluorescein (11426320001, Roche) and subsequently in 1:200 Cy3-conjugated donkey anti-goat IgG and 1:200 Alexa 488-conjugated donkey anti-mouse IgG for 2 h.

For immunohistochemistry, a separated set of floating sections (Table 1) was incubated overnight in 1:5,000 goat anti-PHAL antiserum and 1:5,000 rabbit anti-histidine decarboxylase antiserum (16,045, Progen) and subsequently in 1:400 Alexa 488-conjugated donkey anti-rabbit IgG (Jackson) and 1:200 Cy3-conjugated donkey anti-goat IgG for 2 h, at RT. Sections were rinsed, mounted, and processed for immunofluorescence imaging and data analysis as follows. Specificity controls for anti-rabbit HDC antibody are described in detail by Yu et al. (2019).

### Immunofluorescence imaging and data analysis (PHAL experiments)

For a quick overview, immunofluorescence was observed using a Zeiss Axioplan 2 fluorescence microscope. The images were captured, double exposed while switching filter sets for each fluorochrome, and superimposed in Adobe Photoshop CS4 to create a composite image of the same field as described.

Identification of double-labeled PHAL/TRH-immunofluorescence profiles in the TuLH or double-labeled PHAL/TRH or PHAL/HDC-immunofluorescence fibers in the TMN was determined using a CSU-W1 Yokogawa SDC on an inverted Zeiss microscope Observer Z1, equipped with Plan Neo 20X/0.8 NA and Plan Apo 63X/1.4 N.A. Fluorescence was excited with the 488 nm (50 mW) and 561 nm (20 mW) lines diode lasers and collected with a Bright Line FF01-525/30 nm and 617/73 nm, respectively. Images were acquired with an Andor iXon 5078 controlled with Slide Book 6.17 software. Multiple stage positions were collected using a WK-XYBH-APZ30-AV00FT ASI stage controller and optical sections were collected using a Z-stage ASI Piezo MS- 2000 Controller.

To quantify the degree of colocalization, original images were processed using an ImageJ macro (Schindelin et al. 2012) designed to perform the following procedure. Application of Otsu thresholding (Otsu 1979) over the 488 and 561 channels is to eliminate background pixels. Then, for each single optical section, a set of filtered pixels 488 (Gi) and 561 (Ri) was used to calculate a specific value for the Object Pearson’s coefficient (https://svi.nl/ColocalizationTheory). To illustrate the same set of filtered pixels on each single focal plane, we generated a Pearson colocalization map Mp 3 (https://svi.nl/ColocalizationTheory). A Z-stack obtained from each 488 or 561 thresholded image, with optical cuts of 1 μm size in the Z axis, was merged and represented as a composed image using the green and red channels on the ImageJ software (version 2.1.0/1.53 h, NIH, USA).

### Dual-label fluorescence in situ hybridization

Ten µm (mice) or 18 µm (mice and rat) coronal sections were cut from the rostral extent of the hypothalamic lateral hypothalamus to the tuberal lateral hypothalamus (Paxinos and Watson 2009; Paxinos and Franklin 2001; Table 1) using a Leica CM3050 S cryostat, thaw-mounted on Fisherbrand Superfrost Plus Microscope Slides (Thermo Fisher Scientific; Cat #12-550-15), air-dried, and stored at – 80 °C. The mounted sections were fixed with 4% paraformaldehyde—0.1 M PB (pH 7.4) for 20 min, rinsed in PBS for 5 min, acetylated with 0.25% acetic anhydride in 0.1 M triethanolamine for 10 min, treated with ascending ethanol series (2 min) and chloroform (10 min), partially rehydrated in 95% ethanol, and then processed for in situ hybridization as described (Lazcano et al. 2015; Aguilar-Valles et al. 2007). Four adjacent series (S1–S4) of 18 µm-thick rat coronal sections (n = 3), each containing every 12th section, were hybridized with the mixture of the digoxigenin (dig)-labeled TRH receptor 1 (*Trhr)* (S1, S3) or digoxigenin-labeled TRH receptor 2 (*Trhr2*; S2, S4*)* and either fluorescein-labeled *Trh* or *Hdc* riboprobes. Two series (S1, S2) of 10 µm (*n* = 3) or two series (S1, S2) of 18 µm (*n* = 3) thick mice coronal sections covering the rostro-caudal extent of the TuLH (S1) or the TMN (S2) were hybridized with a mouse digoxigenin-labeled *Trh* and a fluorescein-labeled *Cre* recombinase riboprobe or a dig- labeled *Trh* and a fluorescein-labeled *Hdc* riboprobe. Sections were incubated overnight at 56 °C in a humidified chamber as described (Jones et al. 2019). The specificity of hybridization for each antisense probe has been reported elsewhere using sense probes, which resulted in the absence of specific hybridization signal in the tissues of interest (*Trhr, Trhr2:* Heuer et al. 2000; rat *Trh*: Fekete et al. 2000; mouse *Trh*: Kádár et al. 2010).

The *Trhr*, *Trhr2* and *Trh* probes were detected with peroxidase-conjugated, anti-digoxigenin antibody (diluted 1:100 in 1% blocking reagent, Roche) and the signal was amplified with the Tyramide Signal Amplification (TSA) Plus DIG Kit (Cat# NEL748E001KT, Perkin Elmer) for 30 min, using the DIG amplification reagent at 1:500 dilution in 0.05 M Tris (pH 7.6) containing 0.01% H_2_O_2_.

Sections were then incubated in a rabbit monoclonal anti-digoxigenin antibody (Thermo Fisher, Cat# 700772; at 1 μg/ml concentration) for 3 h in the presence of 2% sodium azide to inactivate peroxidase activity. Sections were thoroughly washed in PBS and incubated overnight in peroxidase-conjugated sheep anti-fluorescein antibody (Roche, Cat# 11426346910; diluted 1:100 in 1% blocking reagent). Signal amplification was applied for 30 min using the TSA Plus Biotin Kit (Perkin Elmer) with the TSA Plus biotin reagent diluted 1:300 in 0.05 M Tris and 0.01% H_2_O_2_. The biotin deposits and the anti-digoxigenin-antibody were detected with Alexa Fluor 488-conjugated Streptavidin and Alexa 594-conjugated anti-rabbit IgG (Jackson Immunoresearch; 1:200), respectively. Sections were rinsed thoroughly in PBS, once in Tris 0.05 M (pH 7.6) and coverslipped with Vectashield antifade mounting medium with DAPI (Vector Laboratories). Immunofluorescence imaging and data analysis were made as described for CTB except that immunofluorescence was observed using a Leica DM 1000 LED fluorescence microscope with an HI PLAN 10X/0.25 or 40X/0.65 objective, and band-pass (BP) filter sets of 340–380/ dichromatic mirror (DM) 400/ barrier low pass (LP) 425; BP 480/40/DM 505/barrier BP 527/30; BP 560/40/DM 595/barrier BP 645/75 for DAPI, Alexa 488, and Alexa 594, respectively.

Images were captured using a digital Firewire camera (DFC450C, Leica) and a Leica Application Suite software. Sections were double exposed while switching filter sets for each fluorochrome and superimposed in Adobe Photoshop CS6 using an iMac computer to create a composite image of the same field.

### Generation of a transgenic mouse line expressing Cre recombinase in TRH-producing cells

A bacterial artificial chromosome (BAC) clone (Clone ID: RP23-295F1, http://bacpac.chori.org), containing Mus musculus Trh genomic sequence along with 118.9 kb of 5’ flanking sequence and 80.5 kb of 3’ flanking sequence of the TRH gene, was used to generate TRH-Cre mice. The Cre recombinase coding sequence was inserted + 65 bp from the translational start site of TRH gene exon 2, along with a polyadenylation cassette and a kanamycin resistance gene flanked by a flippase-recognition target (FRT) cassette, to allow the selection of recombinants containing the insert. A 3.4 kb-long PCR product containing the *mTrh* flanked by the Cre-polyadenylation cassette-kanamycin amplicon was used to remove the template plasmid. Homologous recombination with electrocompetent cells was induced to insert the 3.4 kb PCR product into the BAC RP23-295F1. The FKF cassette was removed and obtention of a Trh–Cre BAC clone confirmed by sequence.

Positive clones were grown on chloramphenicol/ampicillin-resistant plates, scaled into LB antibiotic-resistant media, and purified using a commercially available kit (Nucleobond Bac Maxi Kit, BD Biosciences Clontech). The Trh–Cre BAC DNA was used to obtain transgenic founders by pronuclear microinjection of 0.5–1 ng of circular plasmid into C57BL/6 J oocytes using standard procedures by the Tufts Medical Center Transgenic Core Facility. Genotyping of transgenic mice was performed by standard PCR on genomic DNA isolated from tail snips at weaning using primers against pro-TRH exon 1 (mTRH sense primer: 5ʹ-TAGGCACCTTGGCACCCTGAT-3ʹ) and Cre recombinase protein (Cre antisense primer: 5ʹ-CCTGGTCGAAATCAGTGCGTT-3ʹ). PCR conditions for this reaction were: 94 °C for 2 min, [94 °C for 45 s, 60 °C for 30 s, 72 °C for 1:30 min] × 30 cycles, 72 °C for 10 min, stored at 4 °C. These primers generate a 600 bp band only in transgenic mice. The IL-2 gene was amplified as an internal control; IL-2 sense: 5ʹCTAGGCCACAGAATTGAAATATCT-3ʹ, IL-2 antisense: 5ʹ-GTAGGTGGAAATTCTAGCATCATCC-3ʹ. PCR conditions used for this reaction were [94 °C for 30 s, 60 °C for 1 min, 72 °C for 30 s] × 29 cycles, 72 °C for 2 min, stored at 4 °C. These primers generate a 350 bp band in all animals. Germline transmission was verified by cross-breeding TRH-Cre positive founders with wild-type C57BL/6 J mice. To maintain the TRH-Cre transgenic line, all subsequent crosses were performed using pure C57BL/6 J mice in Boston, MA USA or pure C57BL/6JN in Cuernavaca, Morelos, México.

### Mapping of TuLH *Trh* neurons in *Trh–Cre* transgenic mice transduced with a Cre recombinase-dependent adeno-associated virus (AAV)

*Trh–Cre* mice generated in our laboratories (see above) were bilaterally injected by stereotaxic administration of an AAV carrying a *Cre*-dependent Gq-coupled hM3D DREADD fused with mCherry under the control of human synapsin promoter (AAV-hSyn-DIO-hM3D(Gq)-mCherry; UNC Vector Core Services, Chapel Hill, NC) at a rate of 100 nl /min (1 × 10^9^ Pfu) with a 20 µm external diameter glass capillary and a World Precision injector (Nanoliter 2010 # 4878); Bregma: AP = − 1.94 mm, DV = − 5.4 mm, L = 0.9 mm (*n* = 10 female; *n* = 5 male) (Paxinos and Franklin 2001; Table 1). The scalp was sutured, and the animal placed on a heating pad until full recovery from surgery and then returned to its home cage; buprenorphine (0.03 mg/ kg body weight; s.c.) was administered once a day for 2 days after injection. Six weeks later, to allow the expression of hM3Dq-mCherry in the TuLH, animals were deeply anesthetized with ketamine (100 mg/Kg) and xylazine (10 mg/Kg), perfused transcardially with 10 ml PBS, pH 7.4, followed by 50 ml of 4% paraformaldehyde in 0.1 M PB, pH 7.4 and their brains dissected and cryoprotected by immersion in 30% sucrose-PBS overnight. The brains were sectioned at 20 µm (Table 1) and then collected to verify the position of the injection and the status of TRH neurons by standard double immunohistochemistry as described above. Sections were incubated in mouse pro-TRH_178-200_ antiserum (1:1000) raised in rat (Péterfi et al. 2018) and rabbit red fluorescent protein (RFP) antiserum (1:1000, Cat. # 600-401-379, Rockland) overnight at RT. After washing, sections were incubated in 1:200 Alexa 488-conjugated donkey anti-rat IgG (Jackson Immunoresearch) and 1:200 Alexa 594-conjugated donkey anti-rabbit IgG (Jackson Immunoresearch) for 2 h. Sections were rinsed and mounted as described above. Immunofluorescence was observed using a Leica DM 1000 LED fluorescence microscope as described above. For a general view and a selection of the regions of interest, images were captured using a digital Firewire camera (DFC450C, Leica) and a Leica Application Suite software. Sections were double exposed while switching filter sets for each fluorochrome and superimposed in Adobe Photoshop CS6 using an iMac computer to create a composite image of the same field.

Confirmation of double-labeled mCherry/TRH-immunofluorescence in the TuLH or double-labeling of mCherry/TRH-immunofluorescence fibers in the TMN was determined using a CSU-W1 Yokogawa SDC on an inverted Zeiss microscope Observer Z1 as described above. Fluorescence was excited with the 488 nm (50 mW) and 561 nm (20 mW) lines diode lasers and collected with a BrightLine FF01-525/30 nm and 617/73 nm, respectively. Images were acquired with an Andor iXon 5078 controlled with Slide Book 6.17 software. Multiple stage positions were collected using a WK-XYBH-APZ30-AV00FT ASI stage controller and optical sections were collected using a Z-stage ASI Piezo MS- 2000 Controller. The analysis of colocalization was made as described for PHAL. A Z-stack obtained from each 488 or 561 thresholded images, with optical cuts of 1 μm size in the Z axis was merged and represented as a composed image, pseudo-coloring the green and red images from the ImageJ software (version 2.1.0/1.53 h, NIH, USA) with Adobe Photoshop CS6 to highlight colocalization.

## Results

### Distribution of cell bodies retrogradely labeled with CTB injected into the rat TMN

**Fig. 1:**
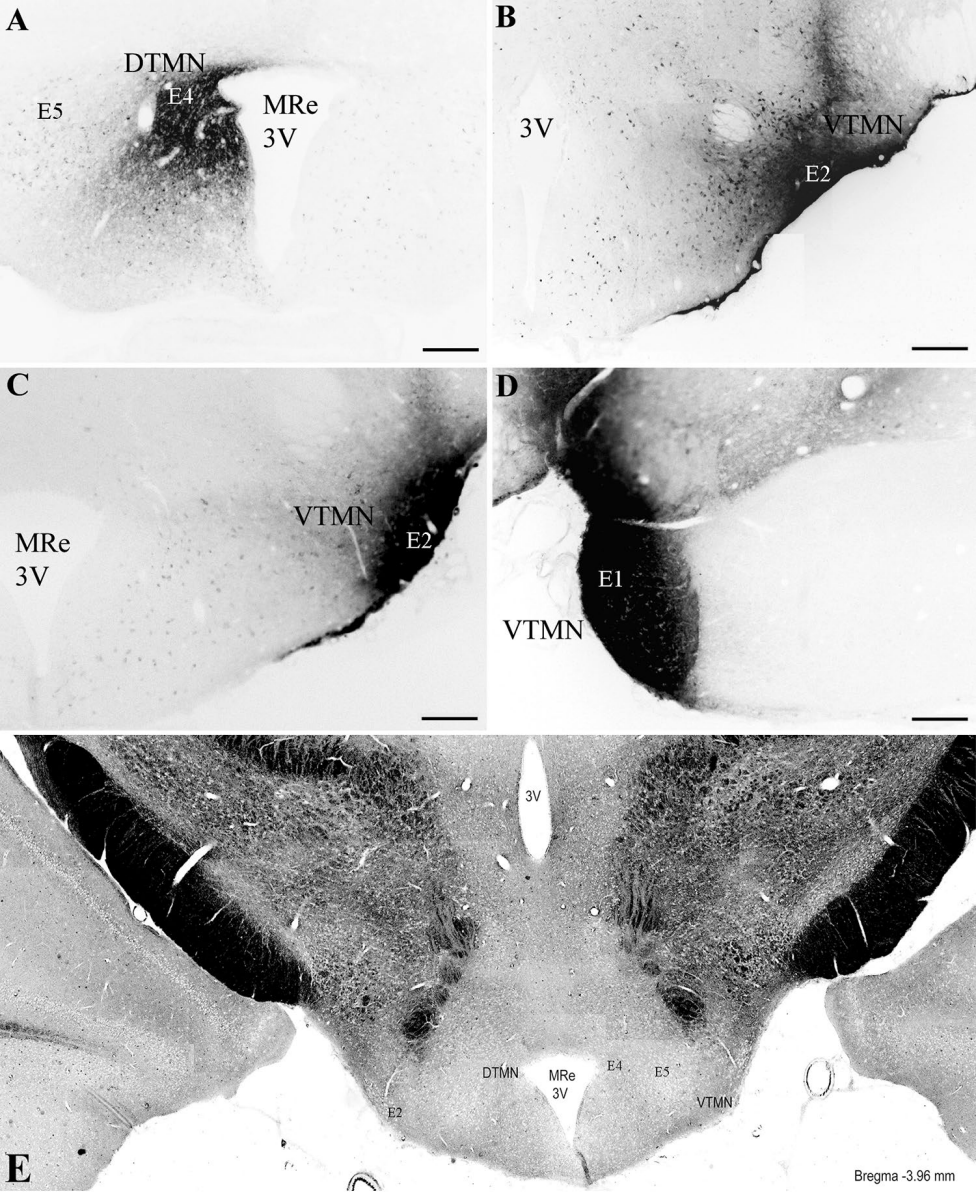
Localization of CTB injection sites in representative rat brains used for the retrograde tracing experiment. **A** CTB core localizes to E4 in the dorsal TMN (DTMN; bregma approx. − 3.72 mm). **B**–**D** CTB cores localize to E1–E2 in the ventral TMN (VTMN; bregma approx. − 3.84 mm to − 4.20 mm). **E** Phase contrast micrography (5 × magnification) shows the position of E4–E5 in the dorsal TMN and of E2 in the ventral TMN. *MRe* mammillary recess; 3 V = third ventricle Scale bar for A–D = 300 µm

The origin of cell bodies projecting to the TMN was identified with the retrograde tracer, CTB. The core injection site covered the dorsal TMN (dTMN, E4–E5 regions) in four of six animals, while the core for the ventral TMN (vTMN, E1–E2 regions) was well positioned in 10 of 19 animals (Table 1). vTMN cores were confined to the border of E1–E2 in 3 animals, diffused slightly from E1–E2 in other 3 animals, and was confined to E1 in 4 animals. Representative CTB injection cores are shown in Fig. 1. After 10 days of transport, CTB neurons were found primarily ipsilateral to the injection sites, with fewer labeled neurons on the contralateral side. CTB afferents to the dTMN and vTMN were visualized in the septum, medial, central and lateral preoptic area (POA), BNST, anterior parvocellular PVN (paPVN), anterior hypothalamus, peduncular part of the lateral hypothalamus (LH), perifornical lateral hypothalamus (PeFLH), tuberal lateral hypothalamus (TuLH), suprachiasmatic nucleus, and medial amygdala.

**Fig. 2:**
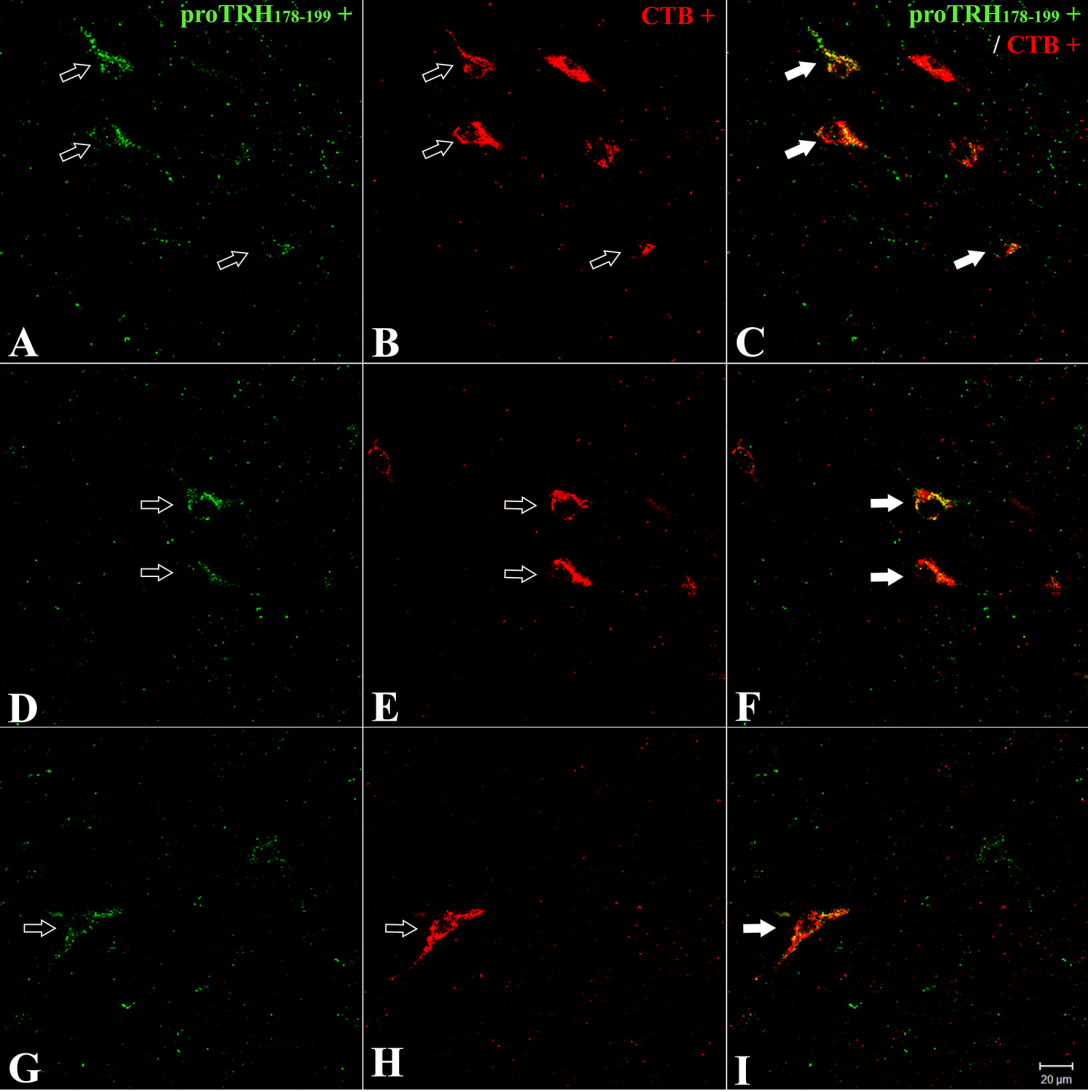
Identification of retrogradely labeled CTB-TRH neurons in the rat tuberal lateral hypothalamus (TuLH). Confocal images from double-labeled immunofluorescent cells demonstrate the presence of CTB (red; empty arrows in **B**, **E**, **H**), retrogradely transported from the ventral TMN, accumulating in TuLH pro-TRH neurons (green; empty arrows in **A**, **D**, **G**). White arrows in **C**, **F**, **I** denote double-labeled cells. Scale bar shown in I denotes magnification in all micrographs

To identify the origin of TRH neurons that innervate the TMN, we conducted dual-label immunofluorescence detection of CTB and pro-TRH_178-199_. Except for the TuLH, none of the nuclei listed above showed colocalization (Fig. 2). The retrogradely labeled TRH neurons from the TuLH were counted in 6 of 14 brains with a successful CTB injection (60 ± 12 TRH cells; Table 1). Colocalization of CTB/pro-TRH immunoreactivity was identified in 64% of TRH cells in 2 rats in which injections were made into the dorsal TMN (E4–E5 regions), 68% of TRH cells in 2 rats in which injections were made into the ventral E1 TMN and 92% of TRH cells in 2 rats in which injections were made into the ventral E2 TMN. However, the number of TuLH-TRH cells targeted with a vTMN E2 injection was statistically different from vTMN E1 or dTMN E4–E5 injection (*P* ≤ 0.001; one-way ANOVA and multiple comparison test Kruskal–Wallis on Ranks).

### Immunolocalization of PHAL/pro-TRH-immunoreactive (IR) axon terminals in the rat TMN

**Fig. 3:**
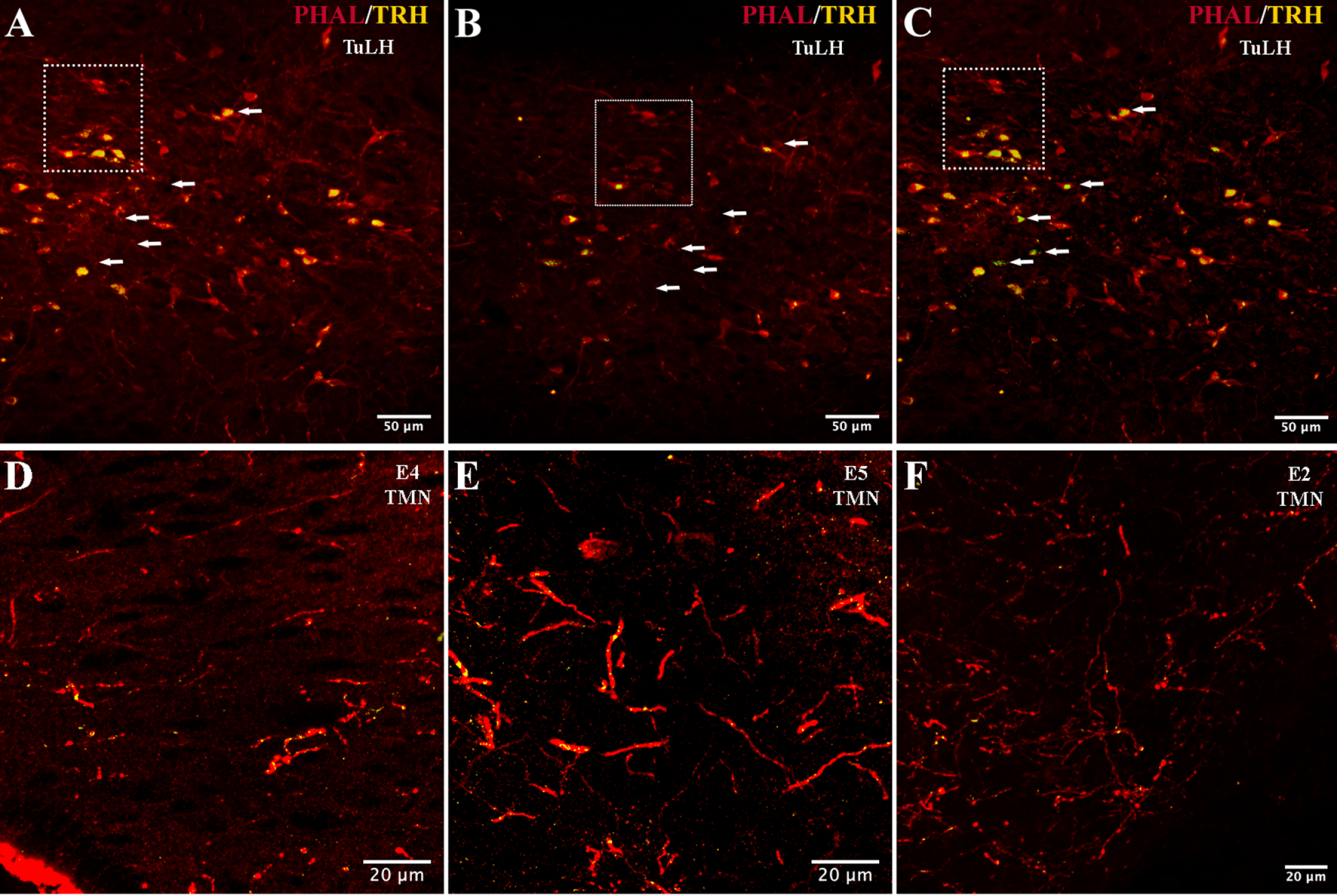
Identification of anterogradely labeled (PHAL injection in the tuberal lateral hypothalamus) PHAL + TRH-IR fibers in the rat TMN. Upper panels indicate immunolocalization of PHAL (yellow) and TRH (red) in the TuLH at − 2.52 mm from the bregma. Two different focal planes for one sample (**A**, **B**) were acquired and the Z projection from the original stacks composed of 26 (**A**) or 32 (**B**) optical slices, each from two channels: 488 (TRH +) and 561 (PHAL +). The resulting projections of each set were merged and represented as a composite image for planes **A** and **B** in **C**. Arrows and inset point to double-labeled cells highlighting in green those which are missing in **A** or **B**. Lower panels demonstrate the presence of pro-TRH in PHAL-containing axon terminals or en-passant boutons from TuLH into dorsal (E4, E5) or ventral (E2) TMN. Each channel of the original stack was processed to eliminate its background using an Otsu thresholding algorithm. Then, given a focal plane, each single voxel of the 561 label (PHAL) channel was analyzed to determine if a counterpart existed in the TRH thresholded channel, such that the referred voxel could be identified as labeled by two marks. The resultant subset was identified as the set of overlapped voxels and using a yellow lookup table. Once the overlapped voxels were identified, the Z-stack was projected using the standard deviation criteria from the Z projection algorithm of Fiji. The resultant image is overleaped with the Z projection of the original set of 561 (PHAL) pixels labeled in yellow. Scale bar is shown on each micrograph

Anterograde PHAL labeling in the TuLH confirmed the specificity of the CTB tract-tracing result. PHAL immunolabeling demonstrated highly discrete injection sites fully covering the TuLH rostro-caudally in 5 of 12 animals and filling dozens of clearly labeled perikarya per section (Fig. 3A–C, Table 1). Acquisition of the same sample in different focal planes allowed us to identify TRH + /PHAL + positive cells, apparently negative for one or the other immunolabeling in one focal plan, in the core (Fig. 3A vs B). PHAL-IR fibers were visualized throughout the hypothalamus, reaching the medial and lateral POA, horizontal limb of diagonal band, periventricular hypothalamic nuclei, ventrolateral hypothalamic nuclei, anterior hypothalamus, PeFLH and peduncular part of the LH, medial BNST, accessory neurosecretory nucleus; anterior, medial and posterior PVN; anterior and lateromedial magnocellular PVN, medial forebrain bundle, paraterete and terete of the hypothalamus, anterior hypothalamus in its posterior part, dorsal and medial arcuate, latero-posterior arcuate; dorsal, central and ventral dorsomedial (DMN) hypothalamus; posterior hypothalamus, mammillothalamic tract, ventral premammillary nuclei and dorsal and ventral TMN. Double-labeled immunofluorescent PHAL/pro-TRH_188-199_ containing axon terminals or en-passant boutons were identified in the dorsal (E4–E5) and ventral (E1–E2) regions of the TMN, with a density of PHAL/pro-TRH immunoreactive fibers low for E4, medium for E5 and high for E2 (Fig. 3D–F).

### Fluorescent in situ hybridization in the TuLH and TMN

**Fig. 4:**
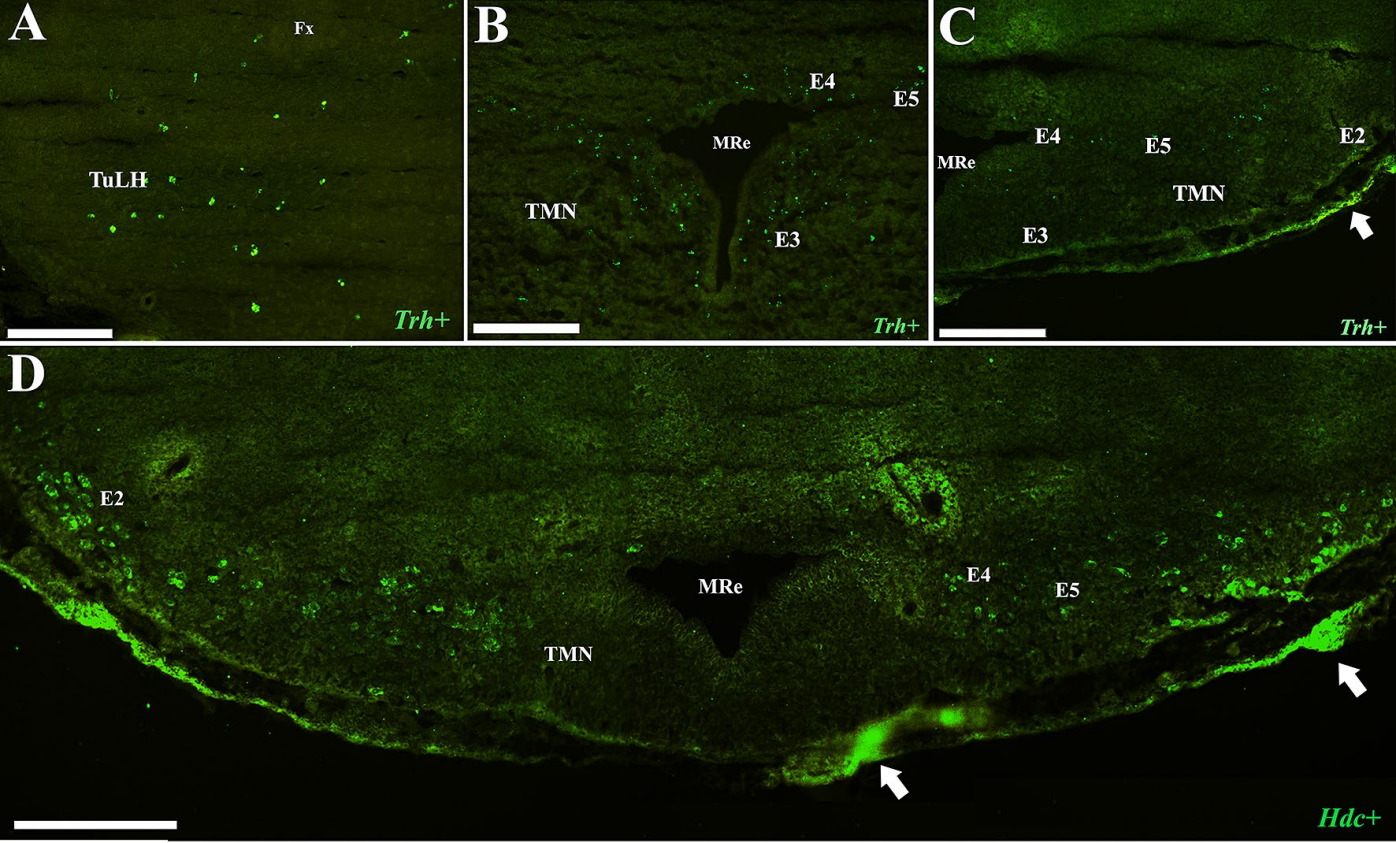
Distribution of *Trh* mRNA in the rat tuberal lateral hypothalamus and *Trh* and *Hdc* mRNA in the rat TMN. **A** *Trh* distribution in the tuberal lateral hypothalamus (green cells, bregma − 2.56 mm) and **B**, **C** in all subdivisions of the TMN (green cells, bregma -3.84 mm). D) Panoramic view of *Hdc* in all subdivisions of TMN (green cells, bregma -3.84 mm). Similar to *Trh* mRNA, *Hdc* mRNA from E1 or E2 accumulates close to the ventral border of the brain, while *Hdc* + or *Trh* + cells from E4 and E5 extend horizontally from the mammillary recess to the E2 subdivision. Note that the fluorescent signal in the arachnoid membrane (arrows in **C** and **D**) is likely due to nonspecific labeling, as specific ISH signals are finely punctate or confluent in the cytoplasm of well-defined individual cells (**A**–**D**). *TuLH* tuberal lateral hypothalamus, *TMN*  tuberomammillary nucleus, *MRe3V* mammillary recess of the third ventricle. Scale bar for **A** = 200 µm; **B, C** = 50 µm; scale bar for **D** = 200 µm

**Fig. 5:**
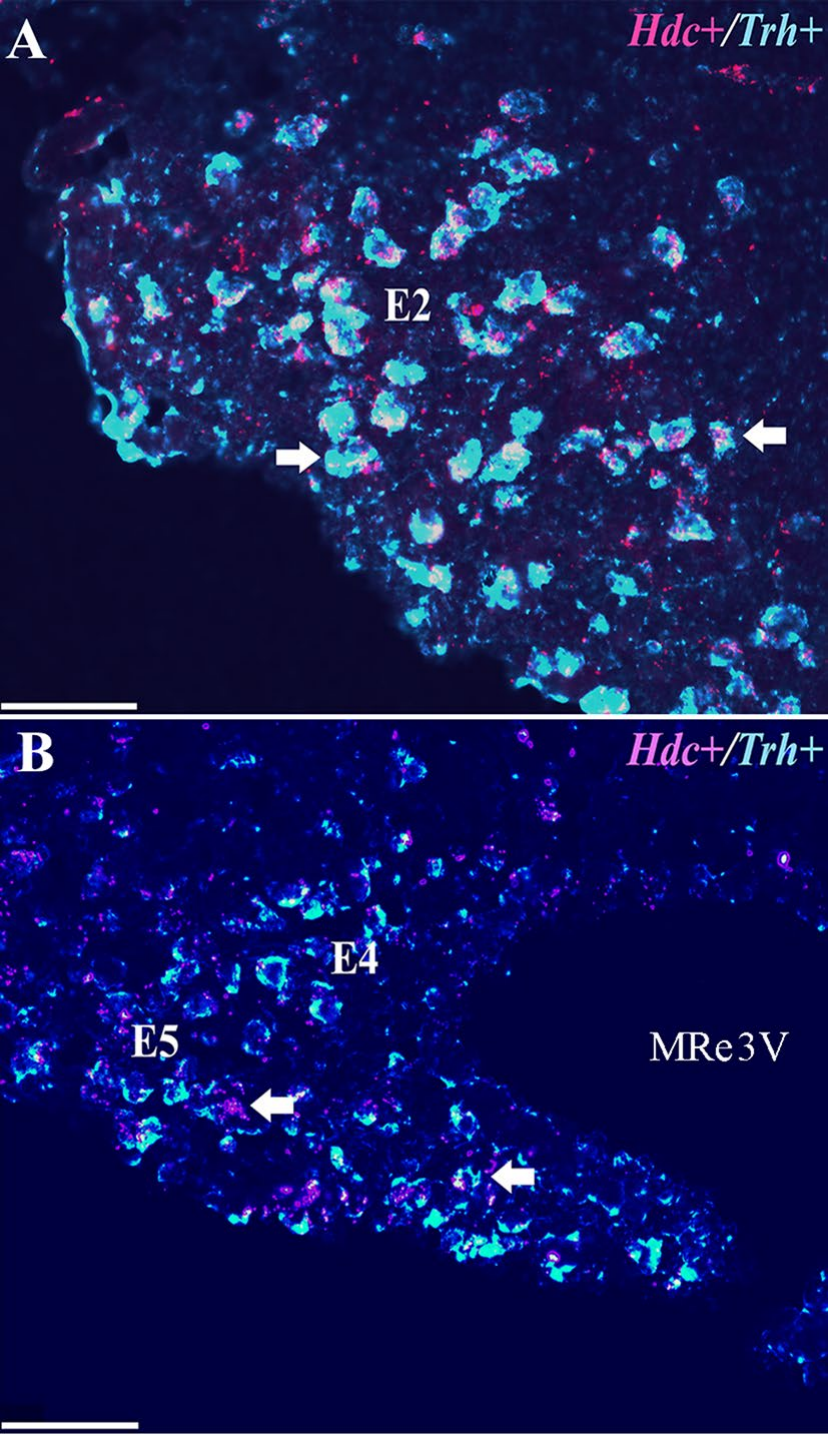
Colocalization of *Hdc* and *Trh* mRNA in the mouse TMN. Double-labeled *Hdc* + and *Trh* + mRNA cells are observed in all subdivisions of TMN. **A** *Hdc* + mRNA (pink) and *Trh* + mRNA (blue) are co-expressed in E1–E2 TMN subdivisions, accumulating close to the ventral border of the brain. **B** Double-labeled *Hdc* + /*Trh* + cells in E4–E5 subdivisions extend horizontally from the mammillary recess toward the E2 *Hdc* + /*Trh* + population. White arrows in denote double-labeled cells. MRe 3 V = mammillary recess of the third ventricle. Scale bar = 50 µm

*Trh* mRNA was mapped in coronal sections containing the TuLH or TMN of adult male rats (*n* = 3; Table 1). Dorso-ventrally, *Trh* mRNA-positive cells in the TuLH were observed below the fornix, between the DMN and the ventromedial (VMN) hypothalamic nuclei (Fig. 4A). *Trh*-positive cells were also observed in the TMN itself, including E3, E4–E5, and along E1–E2 (Fig. 4B, C). *Hdc* mRNA-positive cells showed a distribution pattern similar to that observed for *Trh* mRNA both in the rat (Fig. 4B–D) and the mouse (Fig. 5A, B) TMN. In mice, the *Trh* population represented 46% ± 5 of the TMN cells stained with DAPI (182 ± 6 cells), while histamine cells 39% ± 2. By double-labeling, we observed that 75% ± 7 of *Trh* cells express *Hdc* and 82% ± 5 of *Hdc* cells express *Trh* (Fig. 5A, B).

**Fig. 6:**
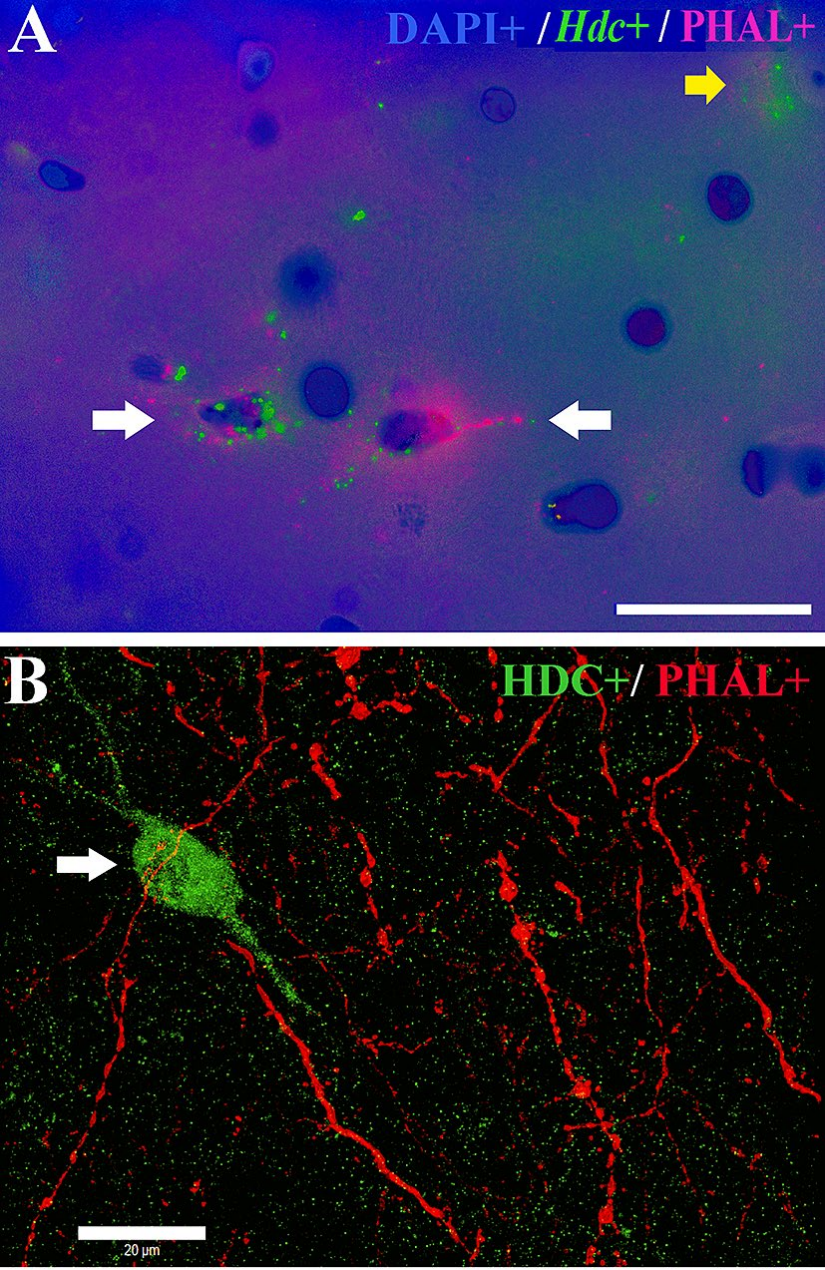
Identification of anterogradely labeled PHAL + fibers on *Hdc* + mRNA or HDC protein cell bodies in the mice TMN. **A** Double-labeling immunolocalization for anti-PHAL (pink) and anti-fluorescein-*Hdc* + (green) mRNA demonstrates the presence of PHAL anterogradely transported from the TuLH to the TMN. Nuclei are contrasted with DAPI (blue). White arrows denote double-labeled cells; the yellow arrow denotes an *Hdc* + cell. Scale bar in A = 20 µm. **B** Confocal images for anti-PHAL (red) anterogradely transported from the TuLH demonstrate the presence of PHAL-containing en-passant boutons on a histamine neuron (green) of the TMN (white arrow)

**Fig. 7:**
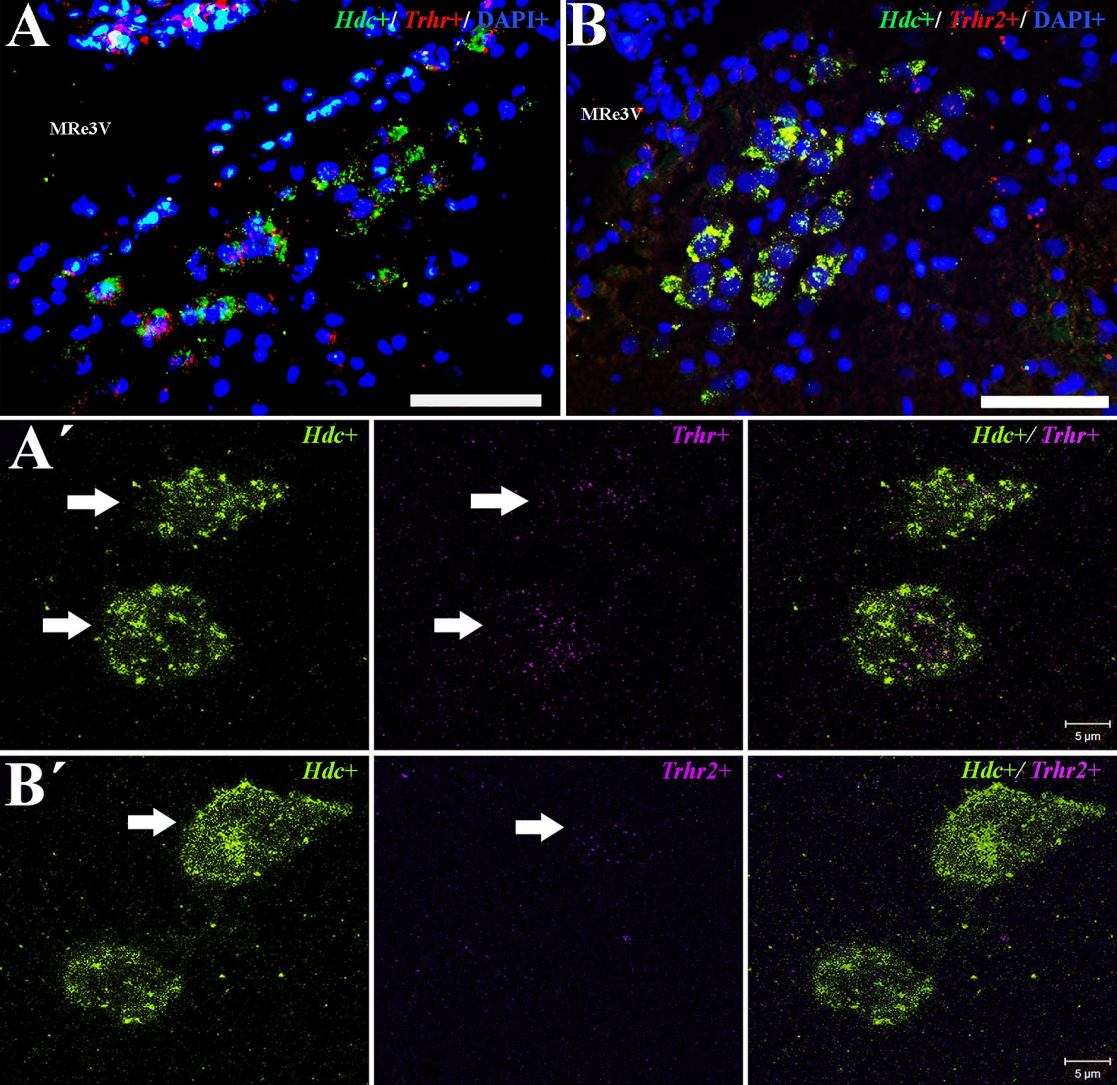
Distribution of *Hdc* mRNA in the rat TMN and its relationship with TRH receptors. *Trhr* is expressed in many *Hdc* + (green signal) cells from the TMN (red signal on **A**), while *Trhr2* expression is low and colocalizes scarcely with *Hdc* + in the TMN (red signal on **B**). Nuclei are contrasted with DAPI (blue). **Aʹ**, **Bʹ** panels show cells that colocalize *Hdc* and *Trhr*, or *Hdc* and *Trhr2*, mRNAs; images are Z-stacks obtained from each fluorochrome in serial optical cuts of 1 μm. White arrows denote double-labeled cells. MRe3V = mammillary recess of the third ventricle. Scale bar for **A, B ** =  50 µm; scale bar for **Aʹ**, **Bʹ** = 5 µm

In addition, *Hdc* mRNA, or immunoreactive HDC, positive cells were reached by immunoreactive PHAL positive fibers whose cell bodies were originated in three of ten animals in the mouse TuLH (Fig. 6A, B; Table 1). Double-labeling for *Hdc* mRNA and *Trhr* or *Trhr2* mRNA in the E4–E5 or E1–E2 subdivisions of rat TMN revealed that 53% of the *Hdc* cells co-labeled with *Trhr* mRNA (30 ± 1 *Hdc* + /*Trhr* + positive cells per field; *n* = 2 fields/ slice/ hemisphere) and 0.6% co-labeled with *Trhr2* mRNA (0.2 ± 0.1 *Hdc* + /*Trhr2* + positive cells per field; *n* = 2 fields/ slice/ hemisphere). Representative images are shown in Fig. 7.

### TRH TuLH-TMN projection in mice

**Fig. 8:**
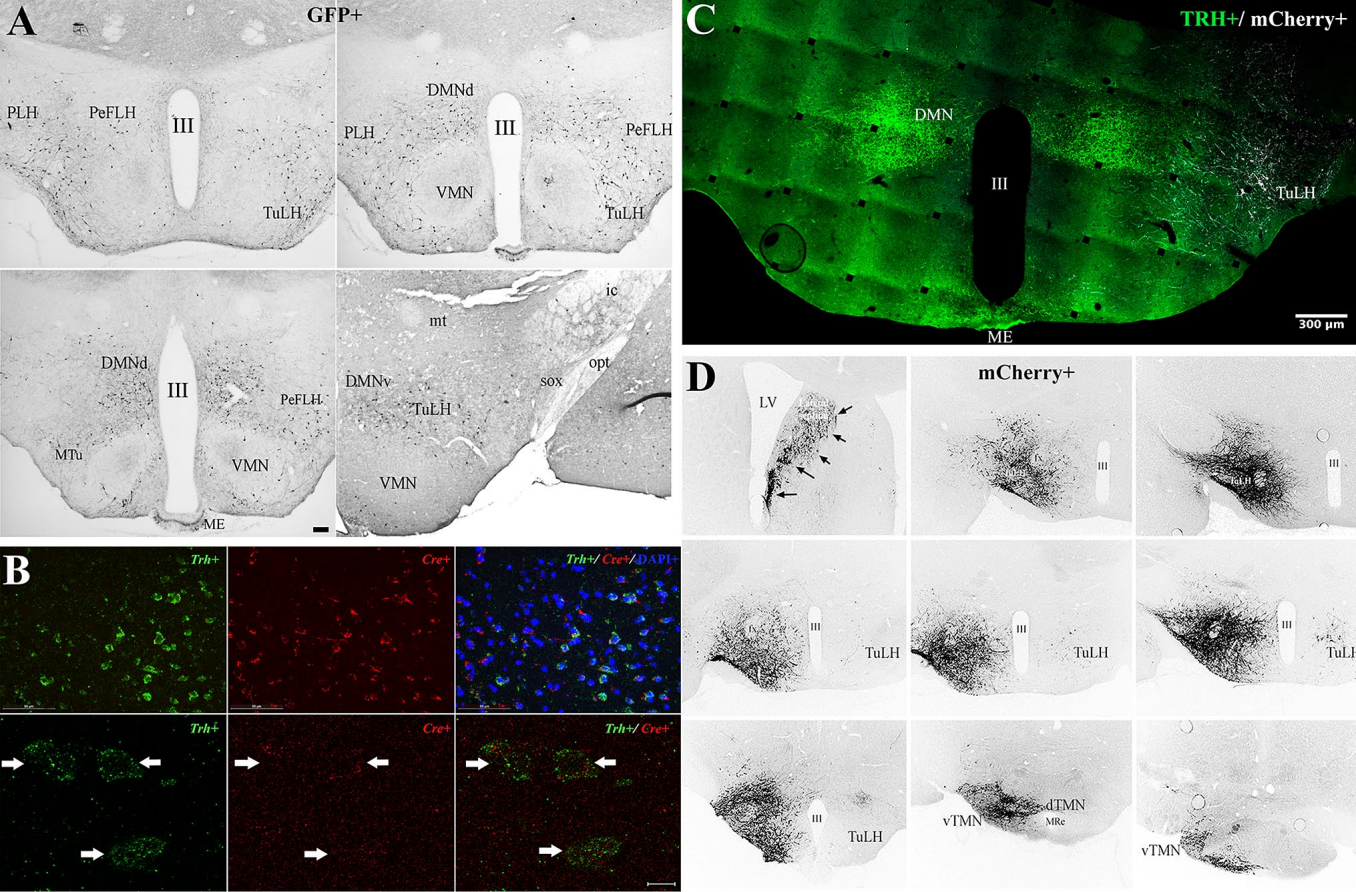
Distribution and activity of *Cre* recombinase in the hypothalamus (TuLH) of TRH-Cre mice. **A** The presence of Cre was mapped in the progeny of TRH-Cre x reporter mice carrying the GFP gene flanked by Lox-P sequences. GFP + cells were detected by immunochemistry in brain slices containing the dorsomedial nuclei and the lateral hypothalamus including perifornical cells and the TuLH, where *Trh* mRNA is expressed. **B** Fluorescent in situ hybridization for *Trh* (green signal) or *Cre* recombinase (red signal) mRNA in 10 μm coronal sections from the TuLH of TRH-Cre male mice. Sections were hybridized with *Trh* digoxigenin-UTP and *Cre* fluorescein- UTP cRNA probes and developed with anti-dig-POD/TSA biotin and Alexa 488 secondary antibody for *Trh* and anti-fluorescein/Cy3 secondary antibodies for *Cre*. Nuclei are contrasted with DAPI (blue signal). White arrows point to *Trh* + or *Cre* + cells obtained from Z-stacks for each fluorochrome at 488 (*Trh*) or 561 (*Cre*) emission channels. **C** Cre-dependent reporter protein mCherry expression is observed 6 weeks after inoculation in the TuLH of a floxed adeno-associated virus (AAV) vector (white signal) but not in the neighboring TRH + cells from the dorsomedial nuclei (DMN; green signal). **D** Immunohistochemistry for mCherry + in TRH-Cre mice inoculated with a floxed-AAV-mCherry in the TuLH allows to easily differentiate cell bodies as well as extensions of mCherry + cells in the rostral and caudal hypothalamus; arrows denote Cre-mCherry positive fibers in the lateral septum. Scale bar on A = 400 µm; B = 50 µm; confocal stacks = 5 µm. *DMNd* dorsomedial hypothalamic nucleus dorsal; *DMNv* dorsomedial hypothalamic nucleus ventral; *ic* internal capsule; *ME* median eminence; *MTu* medial tuberal nucleus; *PeFLH* perifornical part of lateral hypothalamus; *PLH* peduncular part of lateral hypothalamus; *sox* supraoptic decussation; *TuLH* tuberal region of lateral hypothalamus; *LV* lateral ventricle; *dTMN* dorsal tuberomammillary nucleus; *vTMN* ventral tuberomammillary nucleus; *III* 3rd ventricle

*Trh–Cre* transgenic mice were developed in our laboratories (see above) and germline transmission verified and maintained by cross-breeding *Trh–Cre* positive mice with pure C57/BL6/N (Tufts) or C57/BL6/NJ (UNAM) mice per + 10 generations. The expression of green fluorescent protein (GFP) or beta galactosidase (ß-Gal) in *Trh–Cre* positive mice was promoted by crossing the *Trh–Cre* mice with reporter mice Z-EG/GFP, or ROSA 26/Lac Z, flanked by Lox-P sequences (Fig. 8A, Supplemental Figs. S1, S2). The distribution of cells expressing GFP in the CNS was identical to that of *Trh* cells including the olfactory bulb, ventrolateral and medial POA, periventricular hypothalamus, anterior, medial and posterior PVN (Supplemental Figure S1). Figure 8A shows distribution of GFP in the peduncular and perifornical part of the lateral hypothalamus, TuLH, dorsal and ventral DMN, medial tuberal nuclei and arcuate nucleus. Sections from the progeny of the *Trh–Cre* mice hybridized with a fluorescent *Trh* or *Cre* mRNA riboprobe showed an identical distribution pattern in the PVN or thalamus (Supplemental Figure S2A). Double-labeling fluorescent in situ hybridization (FISH) for *Trh* and *Cre* mRNA in 18 µm-thick sections (*n* = 3 male mice; Table 1) in the TuLH revealed 80 ± 4 *Trh*-positive cells or 82 ± 1 *Cre* positive cells corresponding to 18% ± 1 (in each case) of the total number of DAPI nuclei (412 ± 19); furthermore, we detected that 85% ± 2 of *Cre* + cells expressed *Trh*. If FISH was applied to 10 µm-thick sections (*n* = 3 male mice; Table 1), 75 ± 2 *Trh*-positive cells or 72 ± 5 *Cre* positive cells were counted; 93% ± 5 of *Cre* + cells expressed *Trh* (Fig. 8B). Additionally, radioactive ISH for *Trh* mRNA or β-Gal-IR in the progeny of the *Trh–Cre* x ROSA 26/LacZ indicator mice showed that the distribution pattern for β-Gal-immunoreactivity was similar to that of *Trh* neurons (Supplemental Figure S2B,C). Finally, double-labeling immunofluorescence for β-Gal/growth hormone (GH) or β-Gal/thyroid-stimulating hormone (TSH) in the progeny of *Trh–Cre* x ROSA 26/LacZ mice confirmed previous observations of colocalization for TRH in GH-producing cells (corresponding to less than 5% of the GH cell population) and no colocalization in TSH- (Supplemental Figure S2) or adrenocorticotropic hormone-producing cells (not shown).

### Origin of TRH neurons innervating the TMN in *Trh–Cre* transgenic mice

**Fig. 9:**
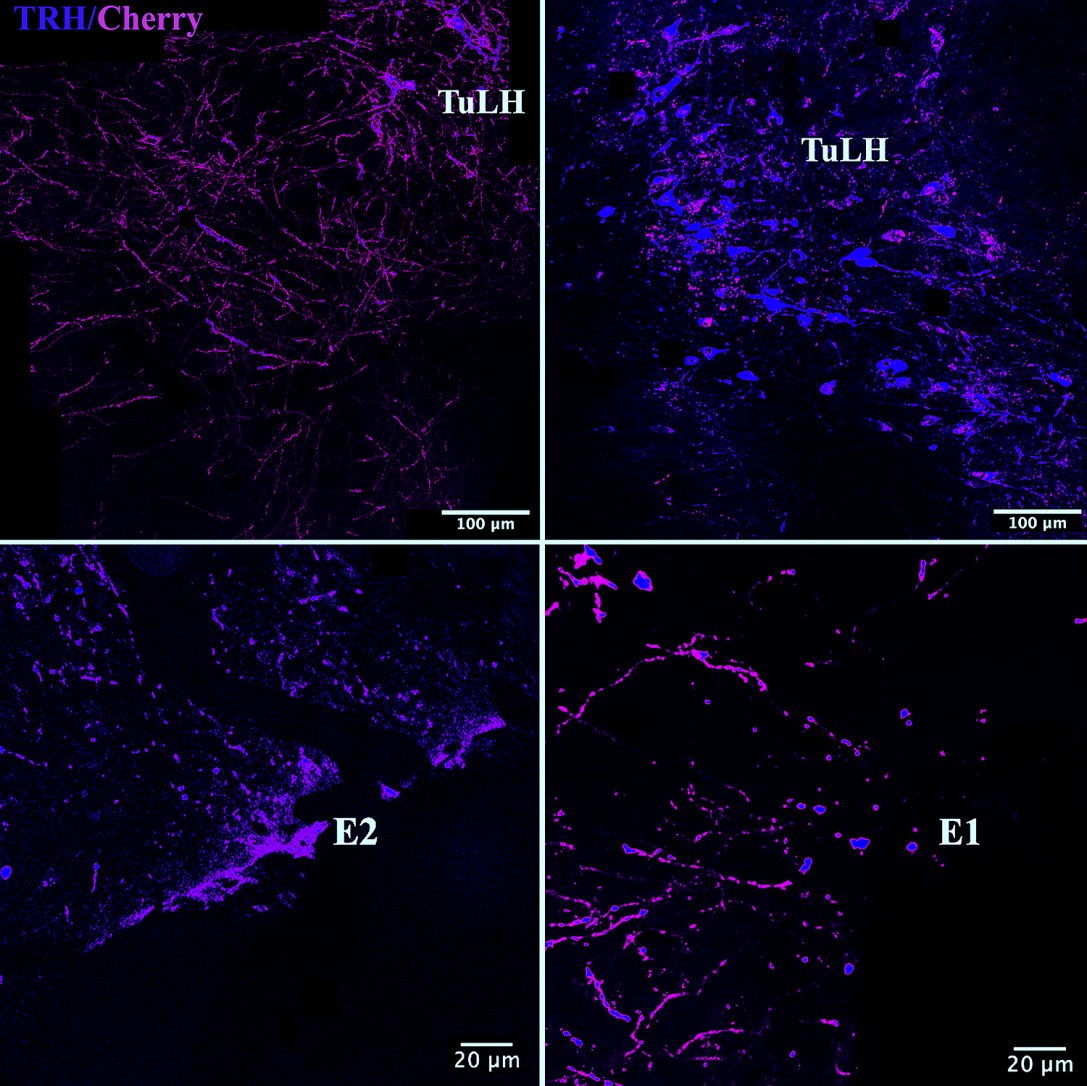
Identification of TMN TRH-IR fibers anterogradely labeled through the intra TuLH injection of a floxed-AAV-mCherry in *Trh–Cre* mice. mCherry was observed, 6 weeks after AAV inoculation, in the tuberal lateral hypothalamus (TuLH) and TMN sub-regions as E1, E2. Cell bodies as well as extensions of mCherry + cells are easily differentiated (upper panels). Lower panels demonstrate the presence of pro-TRH in mCherry-containing axon terminals or en-passant boutons from TuLH into the ventral (E1, E2) TMN. A Z-stack formed by 26 planes (separated by one micron) was acquired using a CSU-W1 Yokogawa SDC on an inverted Zeiss microscope Observer Z1 with a Z-stage ASI Piezo MS- 2000 Controller. Each single plane is composed by two fluorescence channels 488 nm (TRH) and 561 nm (mCherry). The analysis was based on a graphical representation of Manders criteria named Manders colocalization map (Manders et al. 1993). This process starts with a background elimination process performed by mean of an Otsu thresholding algorithm. Then, given a focal plane, each single voxel of the 561 label (mCherry) channel was analyzed to determine if a counterpart existed in the TRH thresholded channel, such that the referred voxel could be identified as labeled by two marks. The resultant subset was identified as the set of overlapped voxels and use of magenta lookup table. Once the overlapped voxels were identified, the Z-Stack was projected using the standard deviation criteria from the Z projection algorithm of Fiji. The resultant image was overleaped with the Z projection of the original set of 561 (mCherry) pixels labeled in red. Scale bar is shown on each micrograph

TRH neuronal projections from the TuLH were mapped in the *Trh–Cre* transgenic mice using a Cre-recombinase–dependent adeno-associated virus (AAV-hSyn-DIO-hM3D(Gq)-mCherry). After 6 weeks of viral transduction, mCherry cell bodies and dendrites were found in the injected side of the TuLH (Fig. 8C). mCherry fibers were also widely distributed in low to medium density in the lateral septum, ventrolateral and medial preoptic area, anterior, medial and posterior parvicellular PVN, dorsal and ventral dorsomedial nucleus, lateral and ventrolateral peduncular part of the hypothalamus and the perifornical part of the lateral hypothalamus surrounding the dorsal and ventral TMN (Fig. 8D). Many double-labeled immunofluorescent mCherry and pro-TRH_188-199_ containing cell bodies and fibers were visualized in the TuLH (Fig. 9). Axon terminals and/or en-passant boutons originating from the virally infected TRH neurons in the TuLH were present in all subdivisions of the TMN, in both sexes (Fig. 9).

## Discussion

Central administration of TRH has a potent anorexigenic effect (Suzuki et al. 1982; Horita 1998), mediated in part through histamine neurons from the TMN (Gotoh et al. 2007). Our data demonstrate that neurons from dorsal and ventral TMN receive inputs from the septum, preoptic area, BNST, PeFx, PVN, anterior and medial PVN, VMN, DMN, peduncular LH and TuLH among others, as previously described (Ericson et al. 1991; Haas et al. 2008), but that TRH cells projecting to the TMN are localized in the TuLH. The origin of the TRH input to the TMN was established by mapping the TRH neurons with two complementary tracers in the rat, and anterograde tracing in transgenic *Trh–cre* mice transduced with a Cre-recombinase-dependent AAV. These studies demonstrate that TMN axon terminals or en-passant boutons containing TRH originate at least in part from the TuLH, but we cannot discard that they may also come from TMN neurons that coexpress *Trh* and *Hdc*. TRH terminals originating in the TuLH may impinge on histaminergic neurons in all subdivisions of the TMN, where approximately half of the histaminergic neurons coexpress *Trhr*.

Histamine neurons are involved in many functions, including alertness, sleep, memory and feeding (Haas and Panula 2003). As these neurons are densely innervated by TRH terminals forming asymmetric synapses (Sarvari et al. 2012), central administration of TRH increases histamine concentration in the TMN (Gotoh et al. 2007) and has a depolarizing effect on histaminergic neurons (Parmentier et al. 2009), we hypothesize that TRH arising from neurons in the TuLH, and possibly from the TMN itself, may mediate some of these responses. TRH neurons of the lateral hypothalamus constitute a large population, distinct from orexin and melanin-concentrating hormone neurons (Horjales-Araujo et al. 2014), but their functional importance is unknown. As TuLH neurons have a predominant glutamatergic phenotype (Ziegler et al. 2002; Stuber and Wise 2016) and the TMN receives excitatory glutamatergic inputs from the lateral hypothalamus (Jang 2014; Yin et al. 2019), co-transmission of TRH/glutamate on some of the histamine neurons may contribute to the excitability of these cells and to physiological arousal. Hypothalamic neuronal histamine regulates the sleep–wake cycle via reciprocal connections to the ventrolateral preoptic area, also known as the ‘‘sleep center’’ and, therefore, activation of the histamine-TRH-R cells by TuLH-TRH/glutamate, and/or possibly TRH/histamine neurons from the TMN itself, may constitute a neuronal network that regulates arousal.

Unfortunately, our efforts to perform triple labeling to show pro-TRH-containing PHAL fibers innervating histaminergic neurons have been unsuccessful, because commercially available histidine decarboxylase antisera are raised in a species that cross-reacts with the other two immunofluorescent labels. However, immunoreactivity of PHAL on *Hdc* mRNA or HDC-IR positive cells in the TMN (Fig. 6A, B) and TMN TRH + /PHAL + fibers originating from cell bodies in the TuLH (Fig. 3) strongly suggest that part of the abundant TRHergic innervation of the histamine neurons comes from the TuLH, and that half of the *Hdc* TMN neurons sense TuLH-TRH inputs, since they express *Trhr*.

The lateral hypothalamus belongs to the reticulate zone of the hypothalamus, containing cell aggregates that lack precise limits. Despite significant progress about the anatomical regionalization and chemical subsets of the LH, differentiation of functional subgroups as mediators of feeding and energy balance responses has remained challenging. Supportive data show that every histologically recognizable subdivision of the LH projects to many nuclei, and although it indicates a lack of specialization, it may be necessary for the integration of behavioral responses. The TuLH, itself, has been divided into at least in six sub-regions: tuber cinereum, medial tuberal nucleus, part 1 or part 2 of the medial tuberal nucleus, dorsal tuberal nucleus, lateral tuberal nucleus and magnocellular tuberal nucleus (Geeraedts et al. 1990; Hahn and Swanson 2010). We observed TRH neurons within the lateral and medial TuLH, confirming earlier studies (Airaksinen et al. 1992; Horjales-Araujo et al. 2014). Collectively, the TuLH establishes broad intrahypothalamic connections, particularly to the medial hypothalamus including the periventricular and perifornical zone, the DMN, anterior and intermediate periventricular nuclei and the parvicellular region of the paraventricular nucleus (Saper et al. 1979; this study). Therefore, it has the potential to strongly influence neuroendocrine control mechanisms.

Studies by Anand and Brobeck (1951) showed that lesioning the TuLH but not the adjacent “satiety center” VMN results in complete absence of spontaneous eating, implicating the TuLH as a responsive nucleus for hunger or the urge to eat. Behavioral responses including a lack of interest even for palatable foods or water persist for several weeks until spontaneous recovery. However, these animals remain leaner than their paired fed controls over their lifespan. While animals with TuLH lesions in the medial zone can recover feeding after injury, ablation of TuLH neurons in the lateral zone produces a more drastic response, as these animals do not survive (Bernardis and Bellinger 1996). Indeed, studies using electrical stimulation of the lateral TuLH have shown that it influences a wide variety of autonomic responses including thermoregulatory, ingestive and affective behaviors (Bernardis and Bellinger 1996) that may contribute to the increased mortality in these animals when lesioned.

Our ISH data indicate that while TRH neurons from the medial TuLH are in a position to influence hypothalamic nuclei related to energy homeostasis, TRH neurons from the lateral TuLH might exert broader effects. *Trh–Cre* AAV mCherry positive perikarya from the TuLH give rise to fibers ascending into the lateral septum, ventrolateral, medial preoptic area, anterior, medial and posterior PVN as well as the dorsal and ventral DMN in addition to descending fibers in the dorsal and ventral TMN (Figs. 8, 9). Furthermore, electrical activity of lateral TuLH neurons gives rise to deep-sleep–wake responses and activity changes related to behavior (Ono et al. 1986). In addition, as the TuLH is traversed by the medial forebrain bundle (Geeraedts et al. 1990), allowing intimate contact with both descending fibers from the basal olfactory, septum, amygdala, nucleus accumbens and, ascending fibers from the brainstem regions, including the ventral tegmental area (VTA), TRH neurons from the TuLH are potentially in a position to participate in the integration of feeding responses as part of the reward and pleasure systems driven by dopamine, GABA and glutamate neurons from the VTA–tuberal LH pathway. Thus, TRH neurons from the TuLH, may function as a relay center in the viscerosensory and visceromotor pathways that impinge on TMN neurons, relaying information onto second order neurons and to the cortex. However, whether the TRH/glutamate TuLH neurons can be activated by hedonic food remains unknown. Furthermore, since TuLH-TRH neurons innervate more fully TMN E2 than TMN E4–E5 neurons, and ventral TMN E1–E2 lesions induce hyperphagia, but dorsal TMN E4–E5 lesions induce polydipsia, independent of food intake (Mahia and Puerto 2006), investigating the relationship between these functional and anatomical differences is warranted.

While most of histamine neurons coexpress GABA, various populations of histaminergic TMN neurons also contain neuropeptides (Haas and Panula 2003). Contrary to Airaksinen et al., who showed that only 14% of histamine neurons were positive for TRH-IR in the rat and none in the mice TMN (Airaksinen et al. 1992), we demonstrated that most of the histamine neurons coexpress *Trh* mRNA in mice (Fig. 5). Assuming that *Trh* mRNA is indeed translated and pro-TRH processing generates TRH in the histamine neurons, differences may arise from fixative methods used by Airaksinen et al. (1992), who needed colchicine and glutaraldehyde fixation to visualize the mature TRH peptide in the rat TMN. An important question is the role of pro-TRH derived peptides in histamine neurons; are they used locally, and/or do they act together with histamine and GABA in their target regions? Histamine and pro-TRH-derived peptides interact functionally in various target regions. Prepro-TRH_178-199_ modulate histaminergic inputs to the PVN that control neuroendocrine outputs (Fukagawa et al. 2007). TRH and histamine interact in arousal mechanisms in the rat nucleus accumbens (Bristow and Bennett 1989). Whether pro-TRH-derived peptides involved in these actions originate in TMN neurons remains unknown.

We conclude that TRH neurons from the TuLH have direct projections to the TMN where they could exert effects on histamine/TRH neurons and potentially other neuronal populations. This interaction is likely of excitatory nature and, may involve the co-release of glutamate. The control of histamine neurons by TRH neurons arising from the TuLH may contribute to the well-known effects of histamine on arousal, energy homeostasis, lipid metabolism and/or thermogenesis.

## Supplementary Information

Below is the link to the electronic supplementary material.

Supplementary file1 Fig. S1 Distribution of a Cre reporter in the CNS of the TRH-Cre mice. The presence of Cre was mapped in the progeny of TRH-Cre x reporter mice carrying the GFP gene flanked by Lox-P sequences. GFP was detected by immunohistochemistry. In the CNS, GFP+ cells are distributed where Trh mRNA is expressed. Scale bar= 400 µm. DMNd, dorsomedial hypothalamic nucleus dorsal; fx, fornix; ic, internal capsule; LV, lateral ventricle; ME, medium eminence; MPOM, medial preoptic nucleus; MRe, mammillary recess of the 3rd ventricle; mt, mammillothalamic tract; MTu, medial tuberal nucleus; OB, olfactory bulb; opt, optic tract; PaAP, paraventricular hypothalamic anterior parvicellular; PaMP, paraventricular hypothalamic medial parvicellular; pe, periventricular hypothalamic nucleus; PeFLH, perifornical part of lateral hypothalamus; pfx, perifornical; PLH, peduncular part of lateral hypothalamus; POA, preoptic area; sox, supraoptic decussation; TuLH, tuberal region of lateral hypothalamus; TMN, tuberomammillary nucleus, dorsal (dTMN) and ventral (vTMN); VLPO, ventrolateral preoptic area; VMN, ventromedial hypothalamic nucleus; III, 3rd ventricle. (TIF 3528 kb)

Supplementary file2 Fig. S2 Distribution and activity of Cre recombinase in the PVN, thalamus and pituitary of TRH-Cre mice. A) Fluorescent in situ hybridization for Trh or Cre recombinase mRNAs in coronal sections containing the thalamus and the hypothalamic paraventricular nucleus (PVN) of C57/BL6JN male mice (left) and TRH-Cre male mice (right); sections were hybridized with digoxigenin-UTP cRNA probes and developed with biotin-tyramide (BT)-AMCA Avidin D. B) Signal distribution for Trh and Cre mRNA shown in A) are compared to that reported for Trh mRNA on the Allen Brain Atlas; arrow in the inset points to the mediodorsal thalamic nucleus (MD). C) Endogenous Trh mRNA was detected with a 35S UTP-labeled cRNA probe in C57/BL6JN male mice (left); immunofluorescence for ß-gal was performed in the offspring of TRH-Cre x ROSA 26 mice (right). Distribution pattern for Trh 35S mRNA in the PVN, was similar to that for Trh developed with BT-AMCA in A) or alkaline phosphatase in B) and Cre mRNA in A) or immunofluorescence for ß-gal in C). Lower panels in C show double-labeling immunofluorescence for ß-gal and TSH in the anterior pituitary demonstrating that Cre expression is excluded from TSH cells, as does that of Trh. Double-labeling immunofluorescence for ß-gal and GH in the anterior pituitary demonstrates that Cre expression occurs in some growth hormone (GH) cells, as expected; double-labeled cells are denoted by the yellow signal. Nuclei in TSH+/B-gal+ and GH+/ B-gal+ are counterstained with DAPI (blue signal). D3V= dorsal 3rd ventricle; AD= anterodorsal thalamic nucleus; sm= stria medullaris of the thalamus; MD= mediodorsal thalamic nucleus, Rt= reticular thalamic nucleus; SFO= subfornical organ; PVN= hypothalamic paraventricular nucleus; III= 3rd ventricle; AP= anterior pituitary. (TIF 12144 kb)

Supplementary file3 Fig. S3 Specificity controls for HDC and PHAL+ fibers in the mice TMN. A, Aʹ) Double labeling immunolocalization of PHAL (red) and HDC (green) in the TMN of a mouse with a PHAL core centered outside the TMN. White arrows denote HDC positive cell bodies (green signal) stained with DAPI (blue signal) in Aʹ. Unlike the well-contoured, well-defined HDC cells, the arachnoid membrane shows nonspecific labeling (pink arrows). B, Bʹ) Excitation for Alexa 488 shows cells positive for HDC (green, white arrows) and excitation for Cy3 shows fibers containing PHAL anterogradely transported from the TuLH to the TMN (red). C1-C3) show sections in which the primary antibody is absent; auto-fluorescence prevails. In Aʹ, Bʹ and C3, nuclei are contrasted with DAPI (blue). Note that nonspecific labeling in the arachnoid membrane disappears when the primary antibody is absent. Scale bar in C1-C3= 200 µm (TIF 9666 kb)

## Data Availability

All raw data are available on demand.
