# Supplementary figures and images for: Origin of thyrotropin-releasing hormone neurons that innervate the tuberomammillary nuclei
Source: Brain Struct Funct. 2022 Aug 7;227(7):2329–47. doi: 10.1007/s00429-022-02527-5 (PMC9418084; doi:10.1007/s00429-022-02527-5)

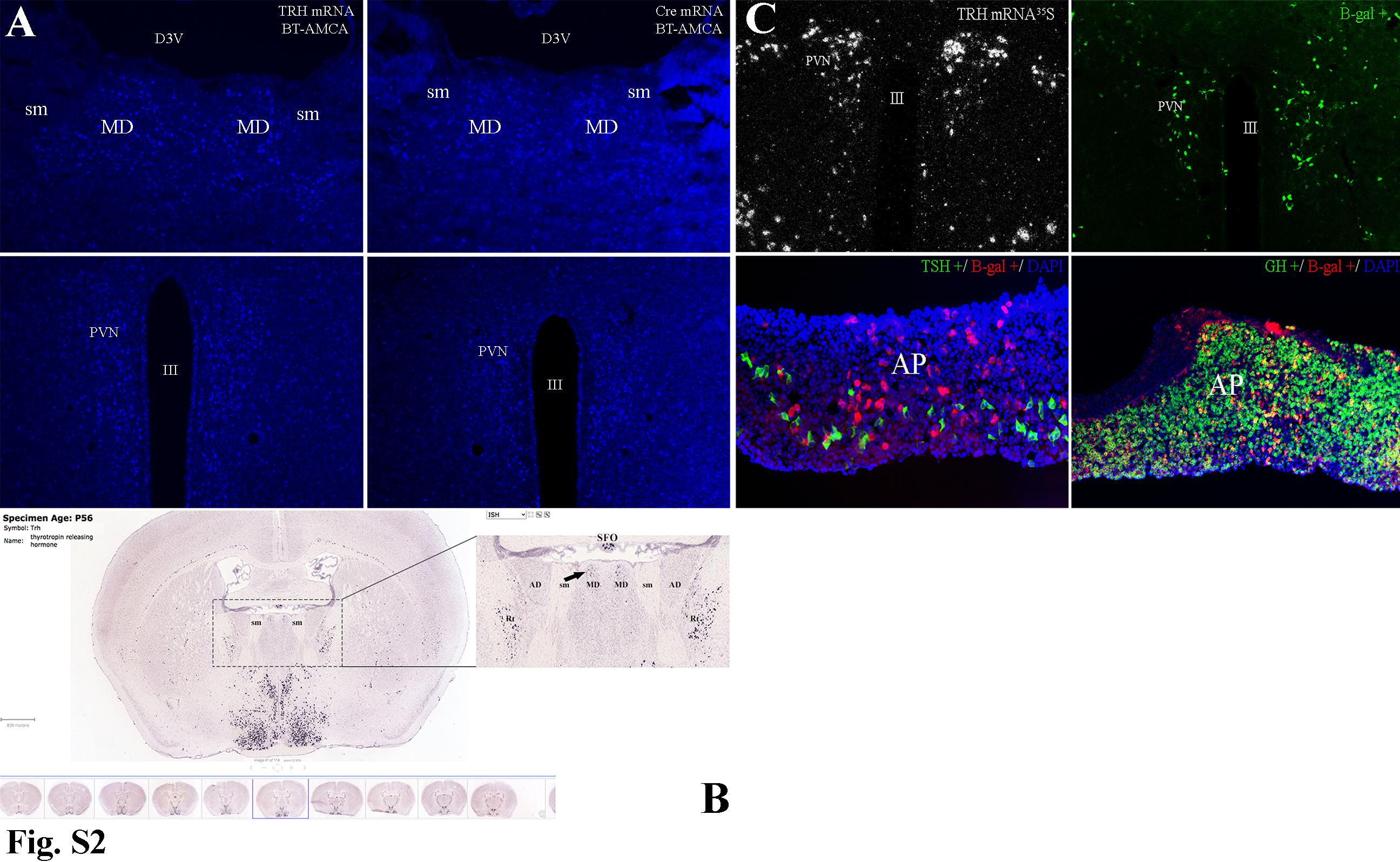

Supplement: Supplementary file 1 — Supplementary file1 Fig. S1 Distribution of a Cre reporter in the CNS of the TRH-Cre mice. The presence of Cre was mapped in the progeny of TRH-Cre x reporter mice carrying the GFP gene flanked by Lox-P sequences. GFP was detected by immunohistochemistry. In the CNS, GFP+ cells are distributed where Trh mRNA is expressed. Scale bar= 400 µm. DMNd, dorsomedial hypothalamic nucleus dorsal; fx, fornix; ic, internal capsule; LV, lateral ventricle; ME, medium eminence; MPOM, medial preoptic nucleus; MRe, mammillary recess of the 3rd ventricle; mt, mammillothalamic tract; MTu, medial tuberal nucleus; OB, olfactory bulb; opt, optic tract; PaAP, paraventricular hypothalamic anterior parvicellular; PaMP, paraventricular hypothalamic medial parvicellular; pe, periventricular hypothalamic nucleus; PeFLH, perifornical part of lateral hypothalamus; pfx, perifornical; PLH, peduncular part of lateral hypothalamus; POA, preoptic area; sox, supraoptic decussation; TuLH, tuberal region of lateral hypothalamus; TMN, tuberomammillary nucleus, dorsal (dTMN) and ventral (vTMN); VLPO, ventrolateral preoptic area; VMN, ventromedial hypothalamic nucleus; III, 3rd ventricle. (TIF 3528 kb) [file 429_2022_2527_MOESM1_ESM.tif]

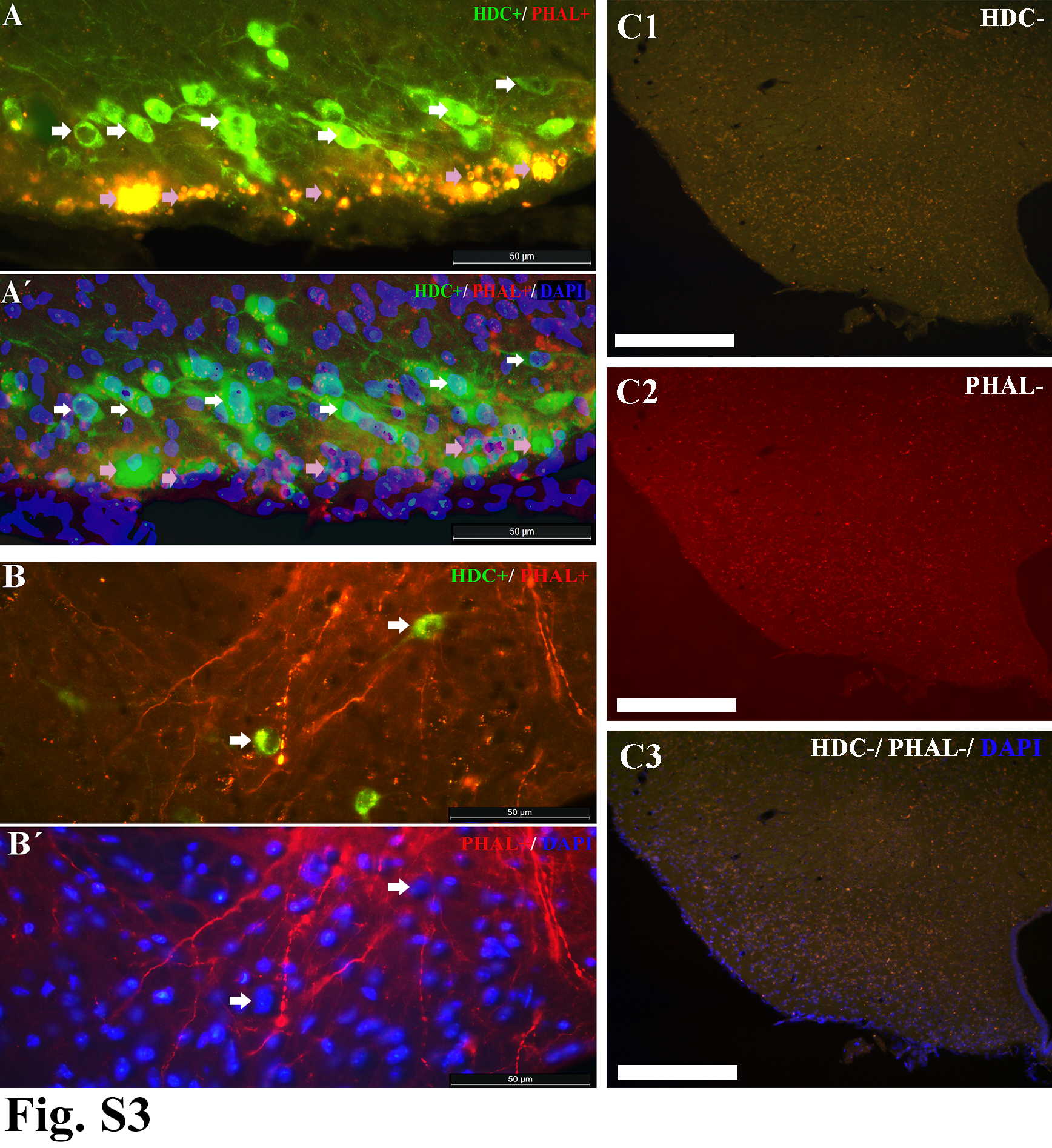

Supplement: Supplementary file 2 — Supplementary file2 Fig. S2 Distribution and activity of Cre recombinase in the PVN, thalamus and pituitary of TRH-Cre mice. A) Fluorescent in situ hybridization for Trh or Cre recombinase mRNAs in coronal sections containing the thalamus and the hypothalamic paraventricular nucleus (PVN) of C57/BL6JN male mice (left) and TRH-Cre male mice (right); sections were hybridized with digoxigenin-UTP cRNA probes and developed with biotin-tyramide (BT)-AMCA Avidin D. B) Signal distribution for Trh and Cre mRNA shown in A) are compared to that reported for Trh mRNA on the Allen Brain Atlas; arrow in the inset points to the mediodorsal thalamic nucleus (MD). C) Endogenous Trh mRNA was detected with a 35S UTP-labeled cRNA probe in C57/BL6JN male mice (left); immunofluorescence for ß-gal was performed in the offspring of TRH-Cre x ROSA 26 mice (right). Distribution pattern for Trh 35S mRNA in the PVN, was similar to that for Trh developed with BT-AMCA in A) or alkaline phosphatase in B) and Cre mRNA in A) or immunofluorescence for ß-gal in C). Lower panels in C show double-labeling immunofluorescence for ß-gal and TSH in the anterior pituitary demonstrating that Cre expression is excluded from TSH cells, as does that of Trh. Double-labeling immunofluorescence for ß-gal and GH in the anterior pituitary demonstrates that Cre expression occurs in some growth hormone (GH) cells, as expected; double-labeled cells are denoted by the yellow signal. Nuclei in TSH+/B-gal+ and GH+/ B-gal+ are counterstained with DAPI (blue signal). D3V= dorsal 3rd ventricle; AD= anterodorsal thalamic nucleus; sm= stria medullaris of the thalamus; MD= mediodorsal thalamic nucleus, Rt= reticular thalamic nucleus; SFO= subfornical organ; PVN= hypothalamic paraventricular nucleus; III= 3rd ventricle; AP= anterior pituitary. (TIF 12144 kb) [file 429_2022_2527_MOESM2_ESM.tif]

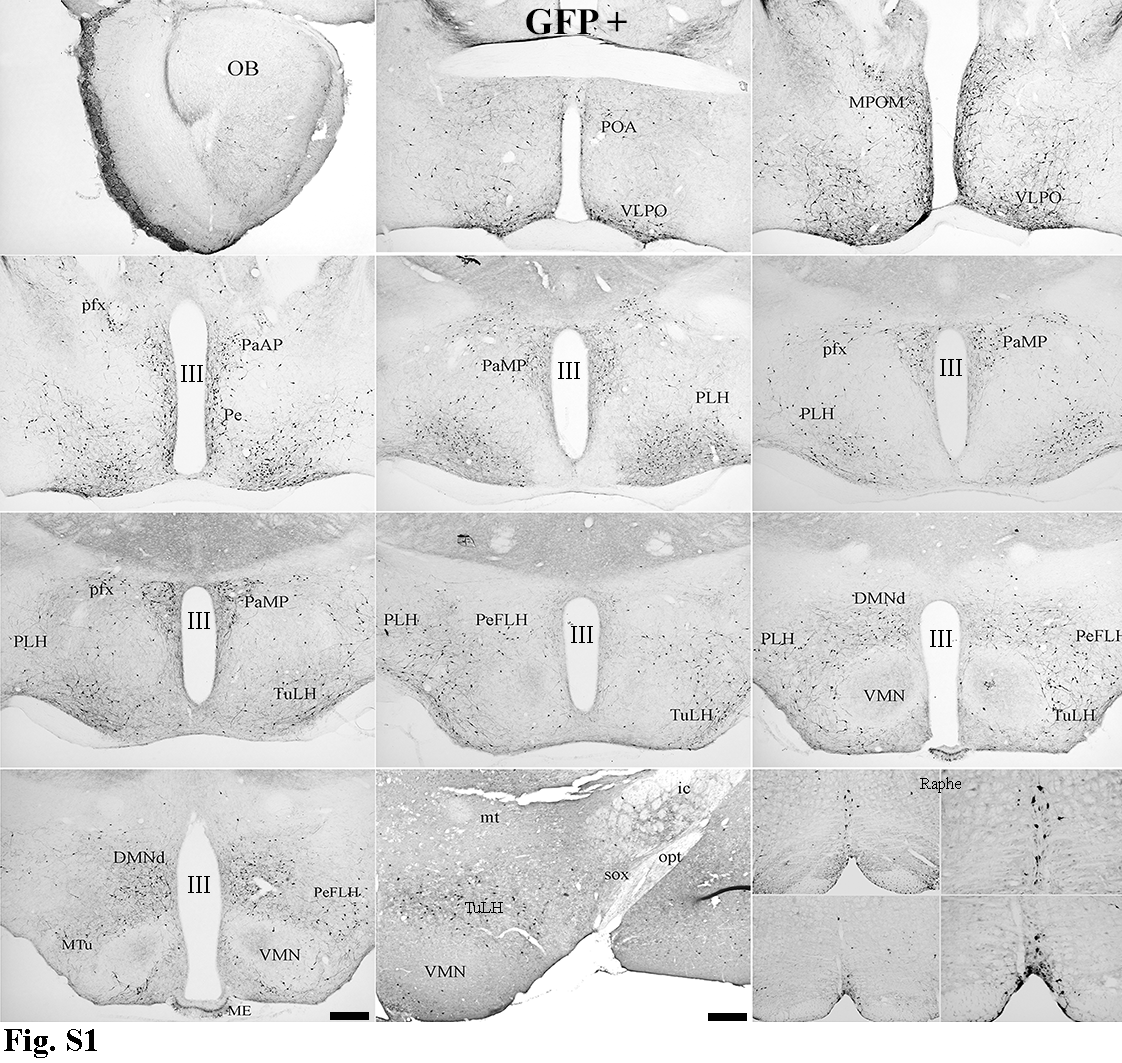

Supplement: Supplementary file 3 — Supplementary file3 Fig. S3 Specificity controls for HDC and PHAL+ fibers in the mice TMN. A, Aʹ) Double labeling immunolocalization of PHAL (red) and HDC (green) in the TMN of a mouse with a PHAL core centered outside the TMN. White arrows denote HDC positive cell bodies (green signal) stained with DAPI (blue signal) in Aʹ. Unlike the well-contoured, well-defined HDC cells, the arachnoid membrane shows nonspecific labeling (pink arrows). B, Bʹ) Excitation for Alexa 488 shows cells positive for HDC (green, white arrows) and excitation for Cy3 shows fibers containing PHAL anterogradely transported from the TuLH to the TMN (red). C1-C3) show sections in which the primary antibody is absent; auto-fluorescence prevails. In Aʹ, Bʹ and C3, nuclei are contrasted with DAPI (blue). Note that nonspecific labeling in the arachnoid membrane disappears when the primary antibody is absent. Scale bar in C1-C3= 200 µm (TIF 9666 kb) [file 429_2022_2527_MOESM3_ESM.tif]
